# Towards fully automated segmentation of rat cardiac MRI by leveraging deep learning frameworks

**DOI:** 10.1038/s41598-022-12378-z

**Published:** 2022-06-02

**Authors:** Daniel Fernández-Llaneza, Andrea Gondová, Harris Vince, Arijit Patra, Magdalena Zurek, Peter Konings, Patrik Kagelid, Leif Hultin

**Affiliations:** 1 Clinical Pharmacology and Safety Sciences, Biopharmaceuticals R&D, AstraZeneca, Pepparedsleden 1, 431 83 Mölndal, SE Sweden; 2 Data Sciences and Quantitative Biology, Discovery Sciences, Biopharmaceuticals R&D, AstraZeneca, Pepparedsleden 1, 431 83 Mölndal, SE Sweden

**Keywords:** Computer science, Scientific data, Statistics, Preclinical research, Magnetic resonance imaging, Cardiovascular diseases

## Abstract

Automated segmentation of human cardiac magnetic resonance datasets has been steadily improving during recent years. Similar applications would be highly useful to improve and speed up the studies of cardiac function in rodents in the preclinical context. However, the transfer of such segmentation methods to the preclinical research is compounded by the limited number of datasets and lower image resolution. In this paper we present a successful application of deep architectures 3D cardiac segmentation for rats in preclinical contexts which to our knowledge has not yet been reported. We developed segmentation models that expand on the standard U-Net architecture and evaluated models separately trained for systole and diastole phases (2MSA) and a single model trained for all phases (1MSA). Furthermore, we calibrated model outputs using a Gaussian process (GP)-based prior to improve phase selection. The resulting models approach human performance in terms of left ventricular segmentation quality and ejection fraction (EF) estimation in both 1MSA and 2MSA settings (Sørensen-Dice score 0.91 ± 0.072 and 0.93 ± 0.032, respectively). 2MSA achieved a mean absolute difference between estimated and reference EF of 3.5 ± 2.5%, while 1MSA resulted in 4.1 ± 3.0%. Applying GPs to 1MSA enabled automating systole and diastole phase selection. Both segmentation approaches (1MSA and 2MSA) were statistically equivalent. Combined with a proposed cardiac phase selection strategy, our work presents an important first step towards a fully automated segmentation pipeline in the context of rat cardiac analysis.

## Introduction

Preclinical assessment of novel cardiovascular therapeutics relies on the use of rodent models. Cardiac magnetic resonance (CMR) imaging is important to this assessment due to its ability to differentiate tissue types with good contrast. Moreover, the non-invasive nature of CMR makes it well suited for a longitudinal follow-up of the same animal during the treatment^1,2^. Changes in volumes of left ventricle (LV) and the ejection fraction (EF) over time are commonly assessed in response to treatment as a proxy estimate of rodent cardiac function.

Currently, the assessment of LV function mainly relies on the manual selection and slice-by-slice segmentation of the ventricle at end-diastole (ED) and end-systole (ES) phases of the cardiac cycle to estimate EF. As the number of animals in a preclinical study can be very large and are followed-up in multiple timepoints, this task can add hours of laborious analysis. Furthermore, the quality of the resulting segmentations depends on experts’ experience and often suffers from intra- and interoperator variability^3–5^. Thus, fully automated segmentation tools to standardise and speed up the segmentation of rodent CMR datasets are highly desirable for in vivo cardiac efficacy studies.

The quality of semi- and fully automated methods for cardiac segmentation of difficult human CMR datasets has been improving thanks to the uptake of convolutional neural networks (CNN)^6–10^. However, there is limited research applied in the preclinical cardiac 3D MRI segmentation sphere in smaller rodent datasets with only two recent examples in mice^6–8^. To our knowledge, this work presents the first statistically robust study into automating the estimation of EF in rats and expands on the well-established 3D U-Net^9,11^ and its derived architectures (i.e., Attention U-Net^12^, U-Net++^13,14^ and V-Net^15^).

Supported by our preliminary work and observations from other teams^12^, LV segmentation of the ES achieves consistently lower performance compared to the ED when attempted by the same model, namely, 1-model segmentation approach (1MSA). As this can lead to undesirable downstream effects on EF estimation, we trained two independent models to segment diastole and systole separately, 2-model segmentation approach (2MSA). Both 1MSA and 2MSA were benchmarked in terms of segmentation quality, robustness against noise and agreement for EF estimates compared to human-derived gold standards. Our results suggest that the presented 2MSA and 1MSA approaches are equivalent in terms of EF estimation and achieve close to human performance which highlights that both 1MSA and 2MSA methods are valid for use depending on the context.

In a similar vein to previous research in the UK Biobank data^16^, the 1MSA approach has been able to segment all cardiac phases captured during the recording of the cardiac cycle. This has allowed us to evaluate the potential of the method for full automation, that is, automated phase selection and EF estimation. Upon probing different metrics derived from LV segmentations along the cardiac cycle (LV volume, LV surface area or mid-slice area), we implemented a GP which identified systole and diastole phases and subsequently estimated EF. This was compared against a conventional fourth-polynomial degree fitting strategy and human selection of systole and diastole. In the light of this evidence, 1MSA emerged as a method with the potential to inspire similar work in other biological systems and organs in the preclinical setting.

## Methods

### Data acquisition

Table 1 summarises the six segmentation datasets used in this study consisting of short-axis 3D CMR images (multi-2D acquisition) from sham-operated rats or rats with induced myocardial infarcts (EF ranging from 41% to 67%). Eleven to thirteen short-axis CINE time series (temporal resolution 8 ms) covering the LV and two perpendicular long-axis slices were acquired per animal. We use the term image stack to refer to the collection of 2D slices which make up a 3D CMR image of the rat LV with a resolution of 0.5 × 0.5 × 1.5 mm. All MRI scanning was performed using a 200 MHz (BioSpec 4.7 T/40 USR; Bruker BioSpin, Karlsruhe, Germany) equipped with a 400 mT/m actively shielded gradient system and ParaVision software (PV6.0). A 72-mm i.d. quadrature resonator (Bruker, Ettlingen, Germany) was used. All experiments were performed in compliance with EU Directive 2010/63/EU and the Animal Research: Reporting of In Vivo Experiments (ARRIVE) guidelines. The protocols were approved by the local ethics committee (Göteborgs djurförsöksetisk nämnd).

**Table 1:** Summary of rat heart longitudinal study image segmentation datasets used in the experiments.

Study name	Number of image stacks	Number of timepoints*	Set
Study 1	126	3	Train
Study 2	73	2	Train
Study 3	43	3	Train
Study 4	92	2	Train
Study 5	116	3	Test
Study 6	94	3	Train(40%)/Test(60%)

*Number of follow-ups within the longitudinal study. In presented studies the timepoints were separated by a 3-month timeframe.

### Pre-processing

LV volumes were manually segmented using the software Segment version 2.2 R6289 (Medviso, http://segment.heiberg.se)^17^ by an experienced operator (Op1) who was considered as the gold standard. Additionally, the dataset Study 5 was segmented by another trained operator (Op2). The operators first cropped the image stack to ease the manual segmentation. All training image stacks and the corresponding masks were resized by interpolation to a standard size of 12 × 86 × 98 voxels and then normalised.

For training, the whole Study 1, Study 2, Study 3 and Study 4 sets were used. Additionally, 38 image stacks from Study 6 were introduced in the training set to increase representativeness of the training set, as this dataset differed from the rest in the extent of cardiac injury of the animal subjects. After training, the model performance was evaluated against the Study 5 and Study 6 test image stacks.

### Implementation details for automated segmentation

In order to achieve automated segmentation, U-Net-derived architectures were leveraged using the manually segmented image stacks described in the previous section.

*Segmentation Approaches Definitions.* Two segmentation approaches for the rat heart cycle were pursued: 1MSA and 2MSA. For 1MSA, one model was trained both on systole and diastole phases and used for segmentation of all phases in the cardiac cycle. In contrast, for 2MSA two independent models were trained separately, namely, one systole and another one for diastole. Thus, this yielded specialised models for systole and diastole segmentation respectively. 2MSA models were not used for segmentation of all phases in the cardiac cycle.

*Model Architectures.* A range of encoder-decoder architectures using CNNs have been implemented, namely, U-Net^9^, Attention U-Net^12^, U-Net++^13^ and V-Net^15^. All the architectures share the common U-Net skeleton, but have added components which aim to increase coherence between the encoding and decoding paths to improve overall pattern recognition. For instance, it was hoped that the attention gates with which Attention U-Nets are equipped would improve the focus of the network on the contours. U-Net++ was used to scope whether an increased connectivity between encoding and decoding pathways through dense skips would lead to better performance. Finally, the use of V-Net was motivated by the fact that this network was specifically designed to process 3D volumes. Further details on the specific characteristics of these architectures can be found in Supplementary Information.

In all architectures, 3D convolutions were applied and the downsampling steps were performed using a volumetric kernel of 2 × 2 × 2 voxels and a stride of 2 × 2 × 2 or 1 × 2 × 2 voxels (depending on the downsampling dimensions). Additionally, 3D padding was applied at the original image to avoid cropping the skip pathways. The padding was set to ‘same’ and the dilation rate was set to 1 × 1 × 1 voxels for the decoding path. The sigmoid was used as the last activation function. Batch normalisation was used after each layer and the dimensions of the tensors were passed as (N, D, H, W, C), where N is the number of sequences (mini batch), D is the number of images in a sequence, H is the height of one image in the sequence, W is the width of one image in the sequence and C is the number of channels (set to 1).

*Training.* A random sample of 10% of the image stacks available for training was separated for validation. Each model was trained for 100 epochs and patience was set to 5 epochs on the validation set. Hyperparameter tuning was performed in a stage-wise fashion: (1) selection of loss function and number of downsampling dimensions, (2) number of blocks and layers, (3) other relevant hyperparameters. Grid search strategy was used for the first stage and random search^19^ for the last two stages. The hyperparameters explored are detailed in Table 2.

**Table 2:** Hyperparameters for model tuning.

Hyperparameter	Value
Loss function	Soft Dice loss^15^, weighted soft Dice loss, binary cross-entropy and hybrid loss (binary cross-entropy + soft Dice loss)
Downsampling dimensions	2 (*x* and *y* axes), 3 (*x*, *y* and *z* axes)
Number of blocks	2,3,4
Number of layers	2,4,6,8
Kernel size	2 × 2 × 2 voxels, 3 × 3 × 3 voxels, 5 × 5 × 5 voxels
Number of filters	8, 16, 32
Batch renormalisation^20^	False, True
Activation function	Exponential Linear Unit (ELU), Gaussian Error Linear Unit (GELU), Scaled Exponential Linear Unit (SELU), swish
Dropout	0.0, 0.2, 0.4
Batch size	8, 16, 32, 64
Optimiser	Adam, RMSProp
Learning rate	0.0001, 0.001
Kernel initialiser	Glorot uniform, Glorot normal, random normal
Pooling	MaxPooling3D (max), AveragePooling3D (average)
Deconvolution	Conv3DTranspose (True), UpSampling3D (False)

The soft Dice loss is formulated as:

1 $${\mathcal{L}}_{DSC} = 1 - \frac{{\mathop \sum \nolimits_{i = 1}^{N} t_{i} p_{i} + \varepsilon }}{{\mathop \sum \nolimits_{i = 1}^{N} t_{i} + p_{i} + \varepsilon }} - \frac{{\mathop \sum \nolimits_{i = 1}^{N} (1 - t_{i} )(1 - p_{i} ) + \varepsilon }}{{\mathop \sum \nolimits_{i = 1}^{N} 2 - t_{i} - p_{i} + \varepsilon }}$$

where *T* is the true foreground segmentation with voxel values *t*_*i*_ and *P* the predicted probabilistic segmentation for the mask over *i* image elements *p*_*i*_. The background class probability is 1 − P. ϵ was set to 1. As a trivial extension, the weighted Dice loss function was designed to penalise misclassification at the borders of the region of interest (ROI). This objective function is given by:

2 $${\mathcal{L}}_{\text{wDSC}}= {\sum_{i = 1}^{N}{\text{w}}_{\text{map}}({\text{t}}_{\text{i}})\mathcal{L}}_{\text{DSC}}$$

The weight map ${\text{w}}_{\text{map}}({\text{t}}_{\text{i}})$ was implemented as described in Ronneberger et al.^9^:

3 $$w_{map} (t_{i} ) = w_{c} (t_{i} ) + w_{0} e^{{\left( { - \frac{{\left( {d_{1} \left( {t_{i} } \right) + d_{2} \left( {t_{i} } \right)} \right)}}{{2\sigma^{2} }}} \right)}}$$

setting *w*_0_ = 2 and *σ* = 1. w_c_ is the weight map to balance the class frequencies, d_1_ denotes the distance to the border of the nearest cell and d_2_ the distance to the border of the second nearest cell.

*Data Augmentation.* One or more of the following augmentation strategies were implemented at random on the original images to improve models’ performance: elastic deformations, random shifts (across the *x*-axis and the *y*-axis), rotation (−20° to 20°), scaling (contraction or expansion), blurring (Gaussian filter with *σ* ϵ [0.5, 1.5]) and gamma correction (γ ~ $\mathcal{N}\left(\text{1,0.1}\right)$). The training dataset was finally inflated by a factor of ten.

*Segmentation Approach Model Optimisation and Selection.* Both for 2MSA and 1MSA we trained four different architectures (i.e., U-Net, U-Net++, Attention U-Net and V-Net) and performed hyperparameter tuning for each of them. For each segmentation approach, the top three best performing architectures were selected based on the Sørensen-Dice score (DSC) on the validation set^21^. Ensembling strategies were also assessed.

Finally, judging the DSC distribution and convergence times, the best performing model for 1MSA was selected. The optimal models for systole and diastole segmentation were selected for 2MSA. Both segmentation approaches were then benchmarked.

### Postprocessing

The results of the segmentation were subjected to thresholding. Subsequently, morphological operations were carried out eroding thin protrusions and closing potential holes within the LV cavity^18^.

### Segmentation approach benchmarking

To substantiate the decision on which segmentation approach would be more suitable for the reliable and robust LV segmentation, 1MSA and 2MSA were benchmarked from three different perspectives: linear mixed models and hypothesis tests, robustness against noise and agreement analysis.

*Statistical Assessment of Segmentation Approaches.* A linear mixed model (LMM) accounting for 1MSA and 2MSA and the two operators using Maximum Likelihood with Satterthwaite degrees of freedom was fit as per Eq. (4)^22^.

4 $${\text{EF}}_{\text{predicted}}={\text{ EF}}_{\text{actual}}+\text{animal\,ID}+{\mathbb{I}}_{\text{segmentation\,approach}}+{\mathbb{I}}_{\text{treatment\,arm}}+{\mathbb{I}}_{\text{operator}}$$

where ${\mathbb{I}}$ denotes an indicator function. ${\mathbb{I}}_{\text{operator}}$ and animal ID are random effects and ${\mathbb{I}}_{ {{\text{segmentation\,approach}}} }$ and ${\mathbb{I}}_{\text{treatment arm}}$ are the model approach and treatment arms fixed effects (see Supplementary Information). Additionally, contrasts were constructed to perform an equivalence test comparing both approaches with an equivalence margin of 2% EF^23^.

*Noise Robustness Analysis.* Previously selected optimal models for 1MSA and 2MSA were then tested in scenarios with increased noise to assess the robustness of their segmentation capabilities. Three test sets were artificially generated based on the images from the Study 5 by introducing Gaussian, Rician^24^ and Rayleigh^25^ distributed noise with a signal to noise ratio (SNR) of 30. Additionally, an extra mixed noise scenario set was generated, where SNR was set to 20 and any of the aforementioned noise distributions was applied to the image at random. DSC was reported, alongside with EF mean absolute differences (MD) both for 1MSA and 2MSA as per Eq. (5):

5 $$MD: = {\mathbb{E}}\left[ {\left| {\Delta EF} \right|} \right] = {\mathbb{E}}\left[ {\left| {EF_{estimated} - EF_{reference} } \right|} \right]$$

*Agreement Analysis*. The main endpoint of preclinical studies is to estimate the EF of a cardiac cycle. Thus, 1MSA and 2MSA models were benchmarked in terms of agreement. Firstly, volumes were thus estimated by calculating the sum of masks for each slice output by each model from 1 and 2MSA. MD and Bland–Altman plots^26^ were used to analyse agreement between reference EF calculated from manual segmentations and the EF estimated from both for 1MSA and 2MSA segmentations.

### Interoperator agreement

We also assessed interoperator variability (Op1 vs Op2) with a Bland–Altman plot for the Study 5 test set. This allowed to evaluate how the proposed automated segmentations compare to the existing interoperator variability.

### Automating 1MSA phase selection for EF estimation

The availability of eleven to thirteen image stacks ordered along the temporal dimension allowed to represent full cardiac rat cycles. We decided to employ the 1MSA model for segmenting all the cardiac phases in each cycle.

First, for a given a cardiac cycle, we explored the optimal way for selecting systole and diastole, as this would impact the final EF estimate. Cardiac volume, slice area and surface area were calculated for each phase to describe the cardiac cycle. The slice area was defined as the 2D slice whose area had the highest variance throughout the cardiac cycle. The surface area was the 2D surface mesh and was calculated using the Lewiner marching cubes algorithm^28^. The temporal ordering of each phase resulted in the cardiac cycle curve which was fit for identifying the systole (local minimum) and the diastole (local maximum). For fitting the curve, two algorithms were compared: a fourth-degree polynomial and a GP model^27^. The prior to fit the GP model to the data was a composite kernel consisting of a constant kernel multiplied by the radial-basis function (RBF) kernel (see Supplementary Information).

The selection of the best metric for selecting systole and diastole was assessed by comparing it with the ground truth. In this case, the ground truth were the systole and diastole phases identified by an experienced operator. To quantify this over all the image stacks, MD and Bland–Altman plots were analysed. Once the optimal metric was identified, the advantages of GP and polynomial fits were discussed to settle which one had the best properties for this use case.

### Software

We implemented the networks in Python v3.7.6, using the Keras library v2.0.8^29^ and TensorFlow v1.14.0 backend^30^. Models were trained on a Tesla K80 using 75 GB of RAM. GPs were fitted using the scikit-learn v0.23.2 and the Lewiner marching cubes used scikit-image v0.17.2. R v3.6.3 was used to calculate intraclass coefficient ICC using irr v0.1.1 and the lme4 v1.1-26 and emmeans v.1.5.2-1 packages for LMM and equivalence testing^31–33^.

### Ethics approval and consent to participate

All experiments were performed in compliance with EU Directive 2010/63/EU. The protocols were approved by the local ethics committee (Göteborgs djurförsöksetisk nämnd).

## Results

### Model optimisation

We assessed the impact of different hyperparameters on the performance of the proposed architectures. The Dice loss is the optimal training loss reaching satisfactory scores in the range between 0.89 and 0.90. Notably, persisting numerical instability issues prevented progress in studying further loss functions (i.e., hybrid loss functions).

The hyperparameters for both the systole and diastole final models from the 2MSA are shown in Tables 3a and 3b, respectively. Table 3c shows the final models from the 1MSA. Further details are provided in Supplementary Information.

**Table 3:** Final Models for Diastole Phase Model for: (**a**) diastole phase model for 2MSA, (**b**) systole phase model for 2MSA, (**c**) 1MSA.

(a) Architecture	Downsampling dimensions	Blocks	Layers	Kernel size	Number of filters	Renormalisation	Activation function	Pooling	Kernel initialiser	Deconvolution	Dropout	Batch size	Learning rate	Optimiser	DSC
Attention U-Net	3	4	6	(3,3,3)	16	True	ELU	average	Glorot normal	True	0.0	8	10^–3^	Adam	0.960
U-Net ++	2	3	8	(3,3,3)	16	True	ELU	max	Glorot normal	True	0.0	8	10^–2^	Adam	0.965
V-Net	2	3	4	(3,3,3)	16	True	ELU	max	Glorot normal	True	0.0	8	10^–2^	Adam	0.959

(b) Architecture	Downsampling dimensions	Blocks	Layers	Kernel size	Number of filters	Renormalisation	Activation function	Pooling	Kernel initialiser	Deconvolution	Dropout	Batch size	Learning rate	Optimiser	DSC
Attention U-Net	3	2	3	(5,5,5)	8	True	Swish	average	Glorot normal	False	0.0	8	10^–2^	Adam	0.929
U-Net	2	5	2	(3,3,3)	16	False	Swish	max	Glorot normal	False	0.2	8	10^–3^	Adam	0.938
V-Net	3	4	6	(3,3,3)	16	True	ELU	max	Glorot normal	True	0.0	8	10^–2^	Adam	0.934

(c) Architecture	Downsampling dimensions	Blocks	Layers	Kernel size	Number of filters	Renormalisation	Activation function	Pooling	Kernel initialiser	Deconvolution	Dropout	Batch size	Learning rate	Optimiser	DSC
Attention U-Net	3	2	3	(3,3,3)	16	True	ELU	max	Glorot normal	True	0.0	8	10^–2^	Adam	0.949
U-Net	2	3	4	(3,3,3)	16	True	ELU	max	Glorot normal	True	0.0	8	10^–2^	Adam	0.948
V-Net	3	3	2	(3,3,3)	16	True	ELU	max	Glorot normal	True	0.0	8	10^–2^	Adam	0.950

The number of downsampling dimensions, blocks and layers have a greater impact on DSC performance than the rest of the hyperparameters. Dropout levels > 0.2 were detrimental across the board and neither GELU nor SELU activation functions contribute to increasing performances compared to ELU. The rest of the hyperparameters (i.e., kernel initialiser, kernel size, number of filters, optimiser, etc.) only cause a marginal improvement in terms of DSC performance and with all of the models converging on the same values.

During the training process, our V-Net implementation converged up to two times faster (33 h for diastole 2MSA) than the rest of the CNN encoder-decoder architectures. In contrast, U-Net++ is the slowest network to train (56 h).

### Model selection

Once the top 3 best-performing models for each segmentation approach were identified, we proceeded to select the optimal one.

**Figure 1:**
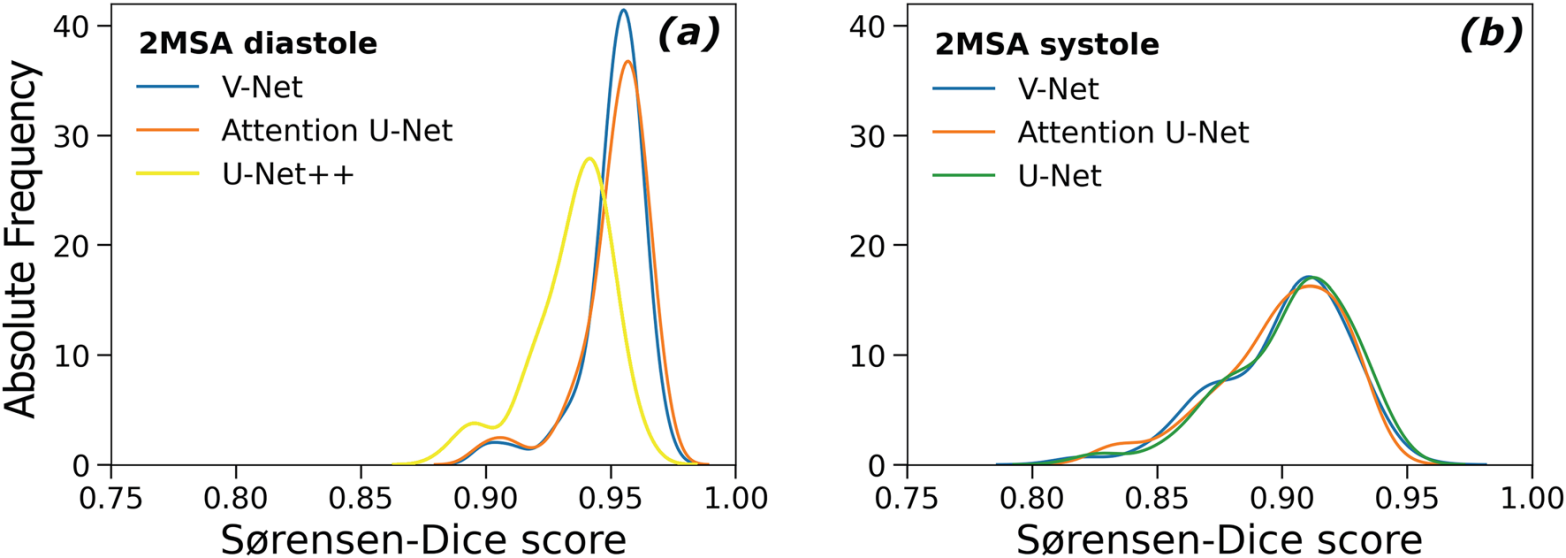
Sørensen-Dice score absolute frequency distribution for 2MSA models for pooled Study 5 and Study 6. (**a**) Diastole models (V-Net: 0.91 ± 0.072, Attention U-Net: 0.91 ± 0.080, U-Net +  + : 0.90 ± 0.069). (**b**) Systole models (V-Net: 0.91 ± 0.072, Attention U-Net: 0.87 ± 0.065, U-Net: 0.87 ± 0.064).

For the 2MSA, all models exhibit a mean DSC > 0.90 and right-skewed distributions (see Fig. 1). Exploratory attempts on ensemble models provided similar if not inferior segmentation statistics.

A comparison between systolic and diastolic segmentation tasks reveals that for 2MSA models, the quality of diastole segmentation was higher for all tested architectures. Given that there was a substantial overlap between DSC distributions for the tested architectures regardless of the phase (see Fig. 1), V-Net was taken as the final segmentation model for systole segmentation thanks to its lower convergence times. Attention U-Net was selected as the optimal model for diastolic segmentation.

**Figure 2:**
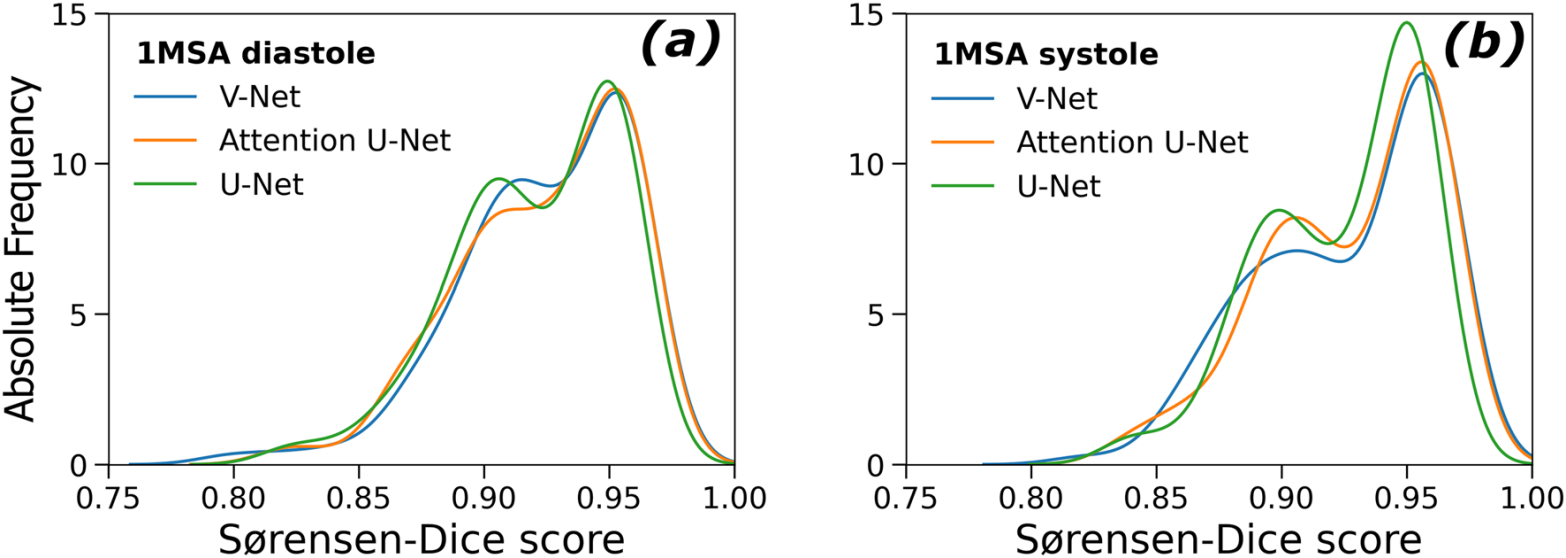
Sørensen-Dice score absolute frequency distribution for 1MSA models for pooled Study 5 and Study 6 where diastole and systole phases were separated manually for the sake of analysing different behaviours. (**a**) Diastole segmentation (V-Net: 0.93 ± 0.032, Attention U-Net: 0.93 ± 0.032, U-Net: 0.93 ± 0.030). (**b**) Systole segmentation (V-Net: 0.93 ± 0.033, Attention U-Net: 0.93 ± 0.031, U-Net: 0.92 ± 0.029).

With regards to 1MSA, performance for base models and ensembles was also comparable. U-Net was selected as the final model on grounds of parsimony (see Fig. 2). Over 75% of the segmented hearts had a DSC > 0.90 regardless of the phase both for 1MSA and 2MSA (see Supplementary Information).

### Segmentation quality analysis

**Figure 3:**
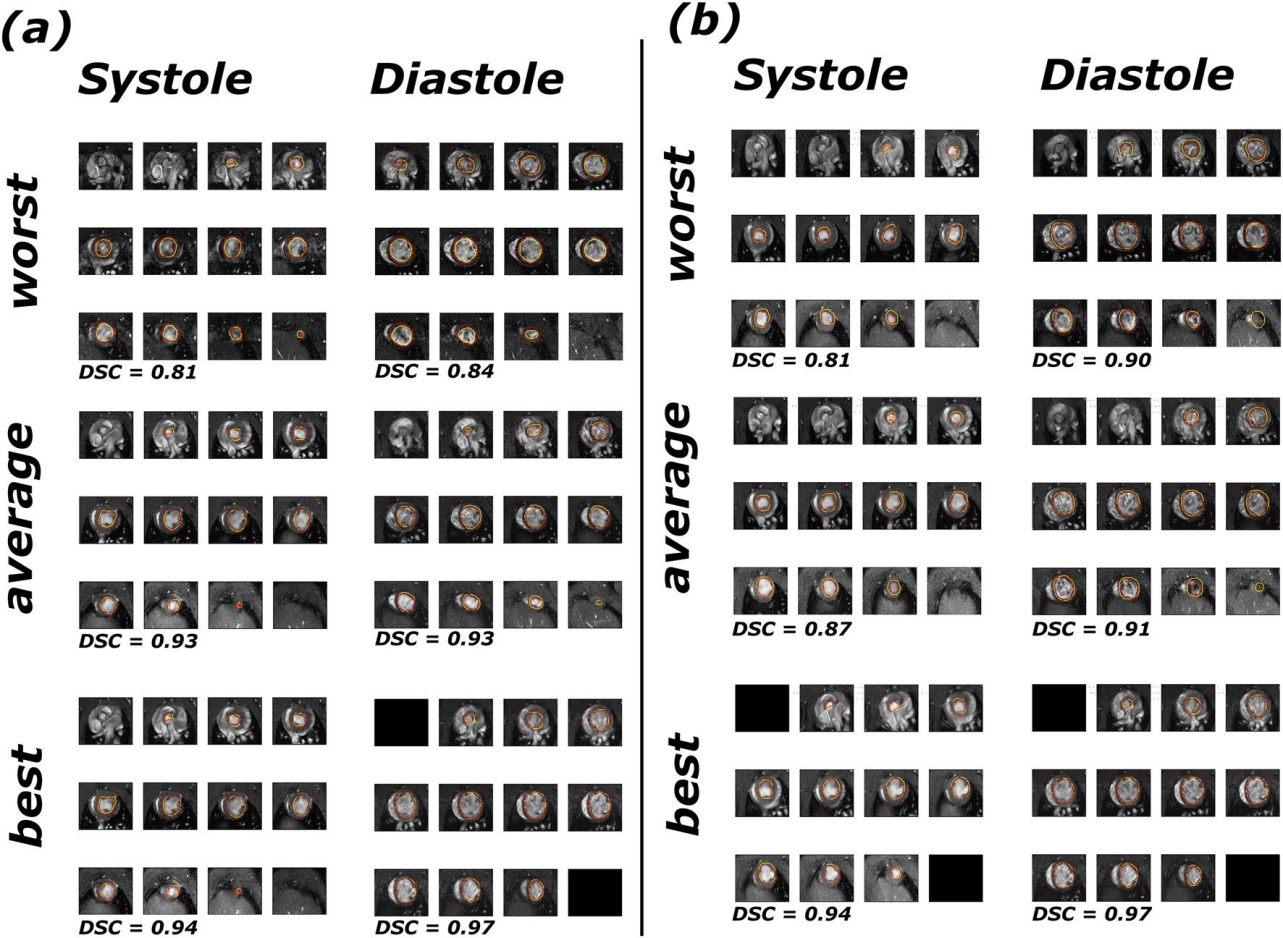
Best, worst and average examples of 1MSA segmented rat hearts with U-Net (systole and diastole) (**a**). Best, worst and average exmaples of 2MSA segmented rat hearts with V-Net (systole), Attention U-Net (diastole) (**b**). In **red** the predicted mask and in **yellow** the ground truth (manual mask).

Representative examples of CNN image stack segmentations from both 1MSA and 2MSA final models are displayed in Fig. 3. From visual inspection of the segmented image stacks, different segmentation methods do not result in substantial differences in segmentation quality. However, systolic contours are consistently more irregular than diastolic contours. Additionally, basal and apical slices suffered from lower segmentation quality compared to midslices (see Supplementary Information).

### Statistical assessment of segmentation approaches

Lastly, the final models both for 1MSA (U-Net) and 2MSA (V-Net for systole and Attention U-Net for diastole) were benchmarked to examine whether significant differences exist between the two approaches.

First, a LMM was fit with the data adjusting for operator and treatment arm (see Table 4). A locally estimated scatterplot smoothing (LOESS) fit was used to aid the interpretation of these differences (see Supplementary Information for diagnostics confirming normality and results). The segmentation of sham-operated and myocardial infarction treatment arms was statistically significantly different and the operator effect (both *p*-values < 0.0001).

**Table 4:** Model table for fixed effects in a linear mixed model.

Term	Estimate	Standard error	Statistic	Degrees of freedom	*p*-value
(Intercept)	8.37	1.96	4.27	3.33	0.019
Human operator	0.87	0.02	47.79	389.54	< 0.0001
Treatment	2.54	0.60	4.20	274.93	< 0.0001
Model	− 1.23	0.22	− 5.48	382.15	< 0.0001

Additionally, we contrasted both approaches based on the LMM with an equivalence test^23^. Results indicate that the null hypothesis of the two approaches not being the same with an equivalence margin of 2% EF could be rejected (*p*-value < 0.001).

Regarding ΔEF MDs, neither segmentation approach was shown superior, given that the absolute differences were in the same order of magnitude (see Table 5).

**Table 5:** Ejection fraction mean absolute difference for pooled Study 5 and Study 6^a^.

ΔEF MD	
2MSA	1MSA
3.5 ± 2.5	4.1 ± 3.0

^a^Represented as MD ± standard deviation.

### Noise robustness analysis

**Figure 4:**
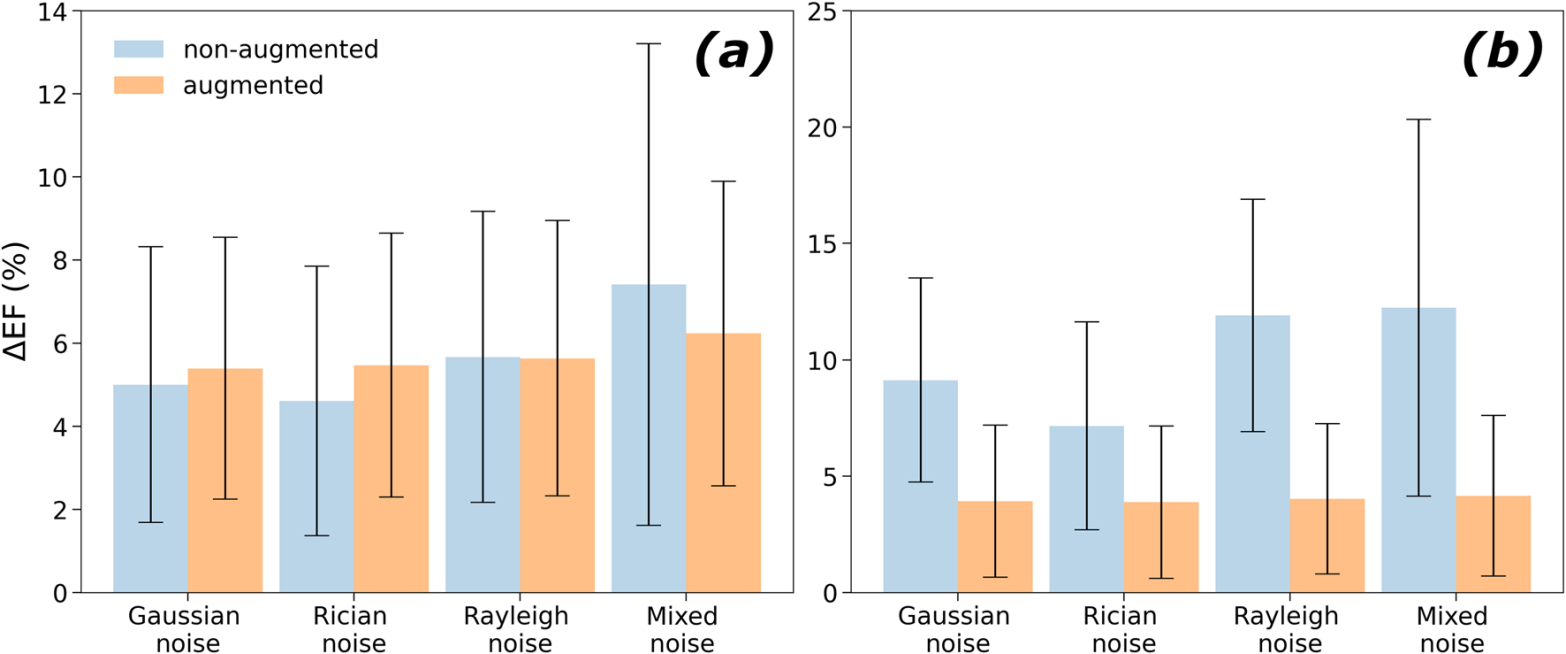
EF mean absolute difference in different noise scenarios between non-augmented (original) and augmented model for one-model segmentation approach (**a**) and two-model segmentation approach (**b**). The bars represent the standard deviation.

In terms of segmentation quality, 2MSA benefits from data augmentation, whereas it does not elicit major improvements for 1MSA (see Fig. 4). The effect of data augmentation on statistics for both segmentation approaches is reported in Supplementary Information.

### Agreement analysis

**Figure 5:**
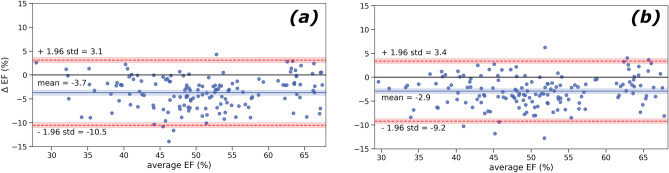
Bland–Altman plots for agreement analysis for pooled Study 5 and Study 6 using the 1MSA (bias =  − 3.7 ± 3.5) (**a**) and 2MSA (bias =  − 2.9 ± 3.2) (**b**). The bias is marked with a solid blue line () and its 95% CI shaded in blue. The equality line is marked with a solid black line (). The upper and lower 95% CI are marked with discontinuous red lines () and are shaded with their respective 95% CI in red.

The agreement between automated and manual segmentation was evaluated to assess whether 1MSA and 2MSA methods were interchangeable. Bland–Altman plots are displayed in Fig. 5 for 1MSA vs Operator 1 and 2MSA vs Operator 2 approaches, respectively. Table 6 contains the main statistics for these plots.

**Table 6:** Bland–Altman plots statistics by segmentation approach for pooled study 5 and study 6^a^.

Segmentation approach	Bias	95% CI upper bound	95% CI lower bound
1MSA	−3.7 ± 3.5	3.1	−10.5
2MSA	−2.9 ± 3.2	3.4	−9.2

^a^Represented as MD ± standard deviation.

Regardless of the segmentation approach, there was a significant negative bias (*p*-value < 0.05). Nevertheless, both 1MSA and 2MSA afforded comparable biases and variability. There was no clear trend upon increasing the magnitude of EF, thereby suggesting that the risk of proportional bias could be rejected.

### Interoperator agreement analysis

**Figure 6:**
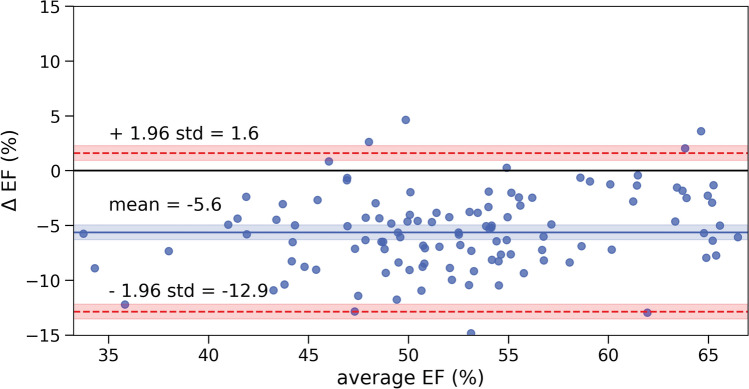
Bland–Altman plot for agreement analysis between operators for Study 5 (bias = -5.6 ± 3.8). The bias is marked with a solid blue line () and its 95% CI shaded in blue. The equality line is marked with a solid black line (). The upper and lower 95% CI are marked with discontinuous red lines () and are shaded with their respective 95% CI.

Interoperator agreement analysis allowed us to set a reference to compare our automation vs manual segmentation level of agreement. The Bland–Altman plot for interoperator agreement is displayed in Fig. 6. The difference between operators is significant, as the equality line is not within the 95% confidence interval (CI) of the mean. The average discrepancy between the two operators is −5.6 ± 3.8%. Interoperator agreement in systole and diastole volume estimation is provided in the Supplementary Information.

### 1MSA phase selection automation for EF estimation feasibility study

The full capabilities of 1MSA could be exploited thanks to the collection of image stacks along the cardiac cycle during the MRI scanning process. Table 7 shows the MD between the EF determined from manually segmented masks and the EF from automatic phase selection for the different selection metrics. Notably, the slice area emerged as the best metric to perform phase selection, as it had the lowest ΔEF MD both for polynomial and GP. In contrast, taking the surface area as the metric for phase selection yields the highest MDs.

**Table 7:** Ejection fraction mean absolute difference comparison for polynomial and GP fitting^a^.

Fitting method	Selection parameter		
	Volume	Surface area	Slice area
Polynomial	8.3 ± 4.7	10.5 ± 3.9	**4.4 ± 4.1**
GP	7.9 ± 4.9	11.3 ± 4.0	**4.7 ± 4.3**

^a^Represented as MD ± standard deviation. In bold, the best performing selection metric.

Agreement both for polynomial and GP using the three selection metrics was also analysed with Bland–Altman plots (see Supplementary Information) and their statistics are summarised in Table 8.

**Table 8:** Bland–Altman plots statistics for phase selection metric^a^.

Metric	Method	Bias	Upper bound	Lower bound
Volume	Polynomial	−11.5 ± 4.3	−3.1	−19.9
	GP	−11.2 ± 4.3	−2.4	−20.0
Surface area	Polynomial	6.7 ± 4.1	14.8	−1.3
	GP	7.4 ± 4.3	15.2	−0.7
**Slice area**	**Polynomial**	**−2.5 ± 4.9**	7.1	−12.1
	**GP**	**−2.5 ± 5.2**	7.3	−12.3

^a^Represented as mean bias ± standard deviation. In bold, the best performing phase selection metric.

A significant bias (*p*-value < 0.05) was observed in all cases. Interestingly, both the volume and the slice area display negative biases, while surface area overestimates the EF. The lowest bias is observed when using the slice area as the phase selection metric and the highest using volume. However, the used fitting method does not substantially impact the bias for any of the metrics.

The difference in absolute terms between the upper and the lower agreement bounds always ranges between 15 and 20. The slice area displays the broader agreement bounds, while the surface area provides the narrowest. No proportional bias is observed and the datapoints are normally distributed.

Taking into account Bland–Altman plot statistics and MD, the slice area is the best metric for selecting systole and diastole phases.

**Figure 7:**
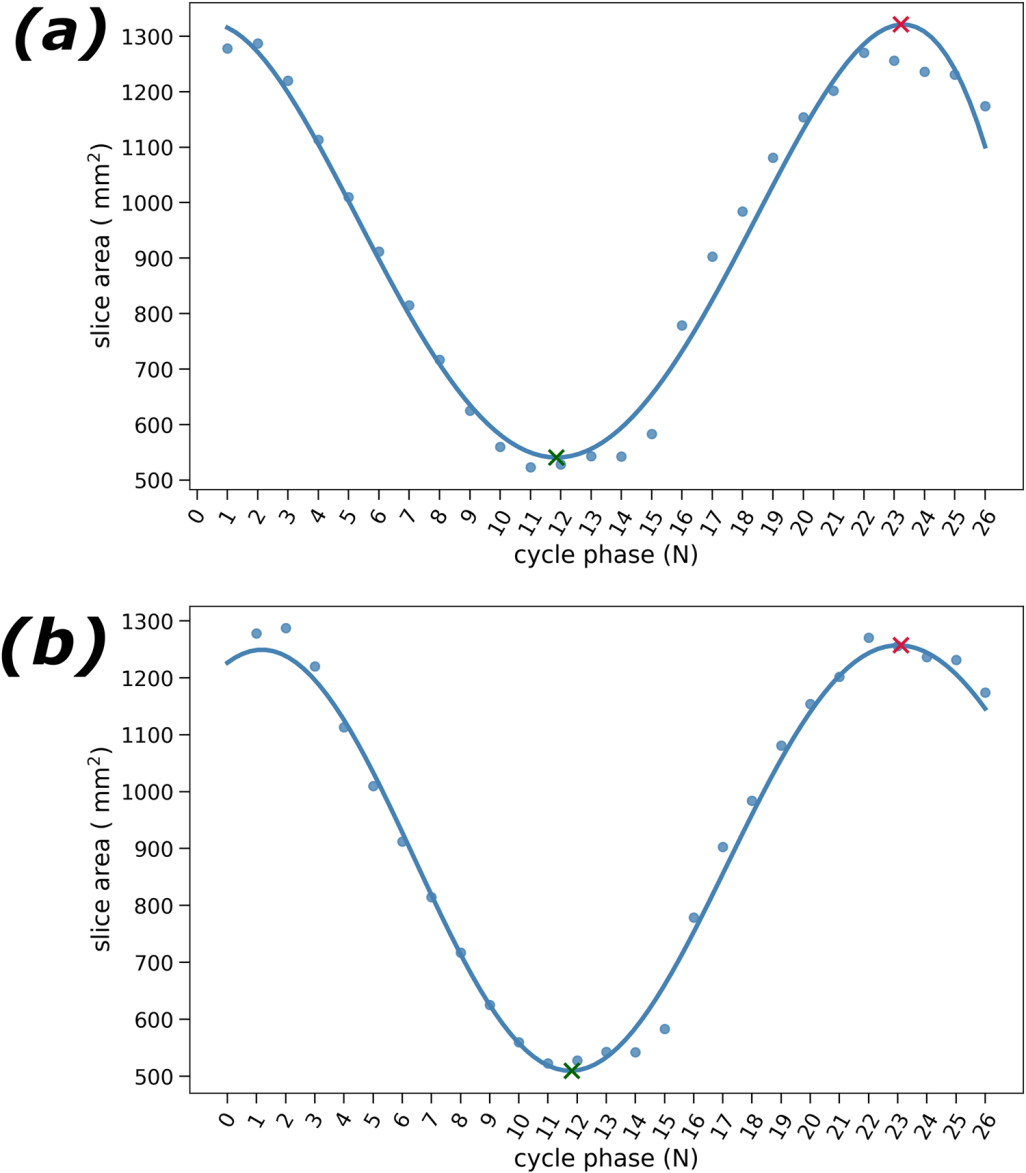
Cardiac cycle plot showing the evolution of the automatically selected slice area vs. the cycle phase.  indicates the selected diastolic phase and  the selected systolic phase. Their volumes are subsequently used to estimate the EF. Fourth-degree polynomial (**a**) and GP (kernel = Constant * RBF) (**b**) fitting methods. The polynomial fit considerably overestimates the end-diastolic volume and does not select an actual datapoint for the end-systolic volume, while the GP fits the datapoints more closely and affords a better estimation of the EF.

The cardiac cycle graphs were visually inspected to determine which method would be better placed to resolve challenging tasks. GP fitting followed the tendency marked by datapoints, whereas the polynomials adopted a generic form which oftentimes ignored finer experimental trends. An archetypical example is depicted in Fig. 7.

## Discussion

U-Net and its derived architectures demonstrated very good performances for rat LV cardiac segmentation. However, the extension of U-Net with attention gates^12^, deep supervision^13^ or residual connections^15^ did not lead to improved performances in our particular context. Our results suggest that simpler U-Nets perform well even in the absence of architectural fine-tuning and extensive skip connections and they remain a highly competitive option for the rat cardiac segmentation. Moreover, U-Nets are capable to successfully extract relevant information even with 2 downsampling dimensions. Against initial expectations^17^, ensembles did not significantly improve performance, possibly due to the correlated nature of their constituent models. This motivated the rationale that model selection should be based on grounds of parsimony, which in its turn reduced convergence speed as more complex architectures (i.e., U-Net++) required longer training times.

**Figure 8:**
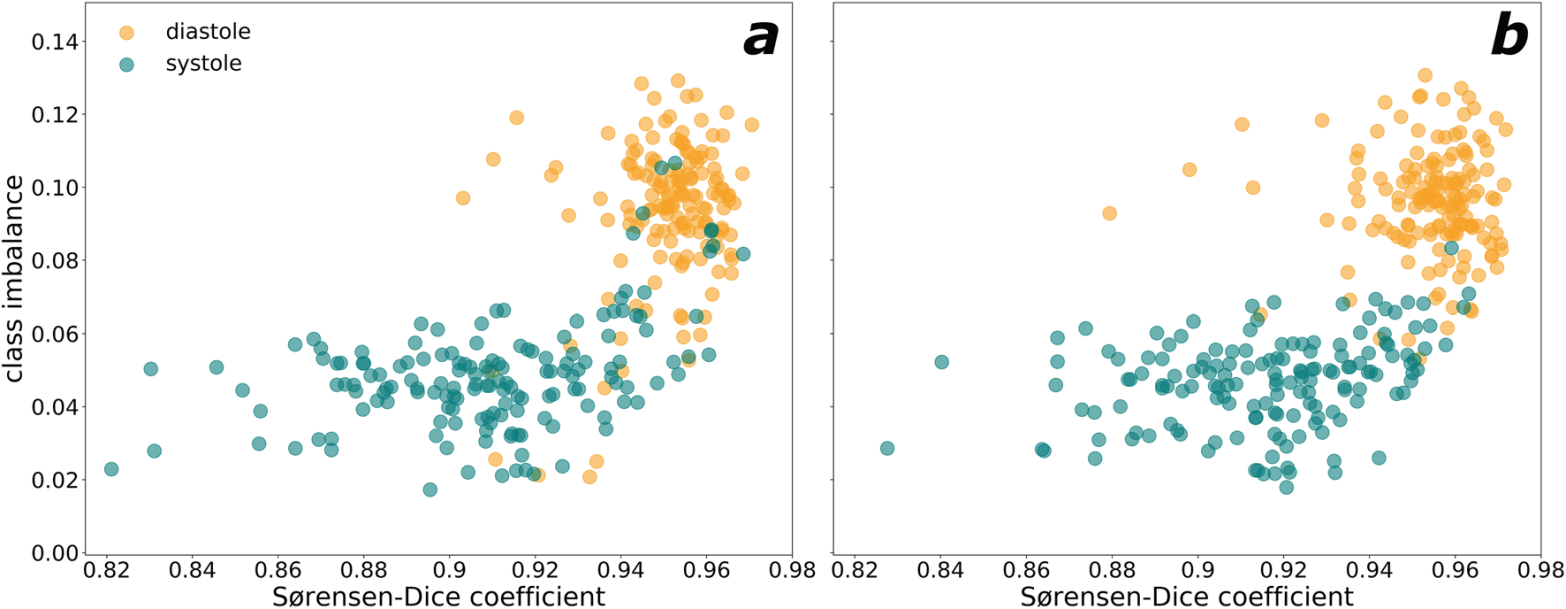
Class imbalance (foreground/background) vs Sørensen-Dice coefficient for systole and diastole phases for pooled Study 5 and Study 6 images with trained V-Net and Attention U-Net from 2MSA (**a**) and U-Net from 1MSA (**b**).

As expected, a factor that influenced the quality of the segmentation is the cardiac phase. The lower segmentation quality in systole could be caused by class imbalance and a higher variability of systole phases compared to diastolic ones (see Fig. 8)^34,35^. This might also be the reason why basal and apical slices suffered from poorer segmentation quality. Increased interslice coherence could play a role in improving segmentation, as it was seen that using 3 downsampling dimensions did not elicit performance improvements. In effect, strategies like leveraging recurrent neural networks have been tried in similar settings^36^ and it could be a potential avenue for future exploration.

The hyperparameter tuning showed moderate performance improvement. Nonetheless, a few trends can be identified. For instance, direct regularisation techniques proved to be detrimental for on our models’ performance. In this respect, it is hypothesised that the application of dropout at random could deprive the model of its most critical training parameters and cause a performance erosion. Our models display consistent performance across two studies and demonstrate robustness against noise. Thus, current evidence does not suggest extensive overfitting. Indeed, these models will be further validated as new studies become available in the future. Interestingly, a substantial drop in DSC was observed whenever renormalisation was not implemented. Finally, the supposed superiority of activation functions with self-normalising properties (i.e., GELU and SELU)^37,38^ was not observed in our context.

Data augmentation has the potential to improve performance^7^, but this was not the case for 1MSA, which could be due to the lack of capacity to incorporate instance variance. Further ablation studies could help to better understand this phenomenon.

In principle, it was expected that the 2MSA would lead to significantly better segmentations than 1MSA. Nevertheless, the LMM fit, the equivalence test and MD analysis revealed that they were equivalent. Both 1MSA and 2MSA demonstrated to model appropriately EFs in the range between 50 and 70%, whilst higher variability was observed outside this region. This likely stems from the natural limitation that the number of available training examples is lower.

The segmentation capability of these models was acceptable when compared to humans, as the bias and limits of agreement in the Bland–Altman plots were satisfactory (detecting an EF change of 5% translates to clinically meaningful results on cardiac performance). Additionally, further agreement analysis revealed that these models held a promise in remediating inter- and intraoperator variability, as well as dramatically reducing the time of analysis. In line with previous efforts^39^, both 1MSA and 2MSA segmentation approaches had a lower bias than between operators and the limits of agreement were comparable. Certainly, these models were substantially quicker (~ 12 s with automated segmentation vs. ~ 20 min with manual segmentation based on *in-house* experience). A shortcoming of this study is the limited number of operators available. The addition of more operators for the assessment of the intraoperator agreement should be explored in the future.

Comparable levels of agreement between 1 and 2MSA prompted further investigation into the benefits of the former approach. Its applicability for automated phase selection leveraging the temporal dimension of each image stack along the cardiac cycle was explored. This approach expands on previous research carried out with 2D human images^40^. Amongst the phase selection metrics, the slice area afforded the lowest bias, potentially due to its sensitivity to small changes. The presence of bias in the selection metrics can be attributed to potential computational artifacts, biases in the data or in the annotation process. The surface area yielded the narrowest limits of agreement, which translates into better precision, as it has lower susceptibility to the noise potentially present in some slices. Thus far, no indication of a selection metric providing both an optimal precision and accuracy has been found. The addition of more rats into the study could help alleviate the bias which the surface area metric suffers from, whilst improving a high variability scenario would be more challenging. Therefore, using the surface area provides more reliable EF estimates than the slice area.

Next, we evaluate which fitting method was best suited to model the cardiac cycle. Visual inspection of the GP and polynomial fits revealed that the GP ability to learn the distribution of datapoints provides the necessary flexibility needed to model the cardiac cycle. Additionally, GPs offer the possibility to develop more advanced approaches such as sparse and variational GP^41^ which can improve further the representation of the cardiac cycle.

The 1MSA provides a seamless phase selection which, coupled with estimation of the EF, allows for a full-automated process. This reduces the segmentation time dramatically and has potential to substantially accelerate pharmacological studies. Whilst 1MSA is beneficial in some contexts, 2MSA can be used in a context where sporadic human intervention is a possibility. It is a simpler approach which can prevent potential errors that might arise from the phase selection process. Thus, the suitability of using either 1MSA or 2MSA would be very much dependant on the specific requirements from the end-user.

Future work will focus around generalising these frameworks for new clinical studies and subsequently improving the predictive capabilities of the existing models by exploring continual learning^42,43^. Another avenue of exploration in the future may be to use the longitudinal data and assess whether this translates into an improvement the segmentation quality. This methodology might inspire further similar applications to other organs and animals of preclinical interest. Indeed, transfer learning can be effectively leveraged for these tasks^44,45^. In this regard, preliminary work indicates that it could be applied successfully in studies with mice hearts.

Our work deals with a fundamental issues of image segmentation in preclinical settings and the success of our models trained on a relatively small preclinical dataset highlights the potential of this technique to be applied to automated cardiac segmentation in preclinical settings.

## Conclusions

We present a successful application of CNNs for the segmentation of rat LV from CMR datasets. Estimates of derived clinical parameters, such as systolic left-ventricular volume, diastolic left-ventricular volume and EF, demonstrate close to human performance in both 2MSA and 1MSA settings. Combined with a novel phase selection strategy based on GPs modelled on the changes of the mid-slice are along the segmented cardiac cycle, our work presents an encouraging first step towards a fully automated rat LV segmentation pipeline. In the future, due to their potential to significantly speed up reporting of results to analysis teams, we hope that such tools will be useful when assessing pharmacological interventions designed to improve cardiac function. As such, we believe that the deep learning based automated segmentation pipeline presented here would inspire research into similar assistive technologies in the preclinical image analysis space in the years to come.

## Data availability

The datasets generated and/or analysed during the current study are not publicly available due to company restrictions but are available from the corresponding author on reasonable request. Code will be made available on request from the corresponding author.

## Supplementary Information

Supplementary Information.

## Abbreviations

1MSA: One-model segmentation approach
2MSA: Two-model segmentation approach
ARRIVE: Animal Research: Reporting of In Vivo Experiments
CI: Confidence interval
CMR: Cardiac magnetic resonance imaging
CNN: Convolutional neural networks
DSC: Sørensen-dice score
ED: End-diastole
EF: Ejection fraction
ES: End-systole
GP: Gaussian process
ICC: Intra-class correlation
LV: Left ventricle
LOESS: Locally estimated scatterplot smoothing
LMM: Linear mixed model
MD: Mean absolute difference (univariate)
RBF: Radial-basis function
ROI: Region of interest
SNR: Signal to noise ratio
