# Supplementary material for: Towards fully automated segmentation of rat cardiac MRI by leveraging deep learning frameworks
Source: Sci Rep. 2022 Jun 2;12:9193. doi: 10.1038/s41598-022-12378-z (PMC9163082; doi:10.1038/s41598-022-12378-z)
Supplement: Supplementary file 1 — Supplementary Information. [file 41598_2022_12378_MOESM1_ESM.docx]

**Supplementary Information**

**Towards Fully Automated Segmentation of Rat Cardiac MRI by Leveraging Deep Learning Frameworks**

Daniel Fernández-Llaneza,^*,†,§^ Andrea Gondová, ^†, §^ Harris Vince,^†^ Arijit Patra,^†^ Magdalena Zurek,^†^ Peter Konings,^‡^ Patrik Kagelid,^†^ Leif Hultin^†^

^†^ Clinical Pharmacology and Safety Sciences, Biopharmaceuticals R&D, AstraZeneca, Pepparedsleden 1, SE 431 83 Mölndal, Sweden

^‡^ Data Sciences & Quantitative Biology, Discovery Sciences, Biopharmaceuticals R&D, AstraZeneca, Pepparedsleden 1, SE 431 83 Mölndal, Sweden

**^§^** DF and AG contributed equally to this work

^*^e-mail: daniel.fernandez1@astrazeneca.com (D. Fernández-Llaneza)

1. **Segmentation Approach Comparison**
   1. **Linear Mixed Model**
      1. **Implementation**

These variables are expressed as indicator functions as such:

$\mathbb{1}_{\text{reader}}\text{(}\text{x}_{\text{i}}\text{)}\text{≔}\left\{ \begin{matrix} \begin{matrix} \text{1} & \text{if }\text{x}_{\text{i}}\text{ }\text{∈}\text{ Op2} \end{matrix} \\ \begin{matrix} \text{0} & \text{if }\text{x}_{\text{i}}\text{ } \end{matrix}\text{∈}\text{ Op1} \end{matrix} \right.$

$\mathbb{1}_{\text{modelling approach}}\text{(}\text{x}_{\text{i}}\text{)}\text{≔}\left\{ \begin{matrix} \begin{matrix} \text{1} & \text{if }\text{x}_{\text{i}}\text{ }\text{∈}\text{ 2MSA} \end{matrix} \\ \begin{matrix} \text{0} & \text{if }\text{x}_{\text{i}}\text{ } \end{matrix}\text{∈}\text{ 1MSA} \end{matrix} \right.$

$\mathbb{1}_{\text{group}}\text{(}\text{x}_{\text{i}}\text{)}\text{≔}\left\{ \begin{aligned} \begin{matrix} \text{1} & \text{if }\text{x}_{\text{i}}\text{ }\text{∈}\text{ na}\text{ï}\text{ve} \end{matrix} \\ \begin{matrix} \text{0} & \text{if }\text{x}_{\text{i}}\text{ } \end{matrix}\text{∈}\text{ myocardial infarction} \end{aligned} \right.$ Equation S1

- - 1. **Mixed Linear Model Diagnostics**

The QQ plot corroborates that the mixed linear model residuals are normally distributed, although there is some deviation from the expected normal line towards the tails which could indicate kurtosis. Nonetheless, the residual distribution is good enough, so the assumption of normality is tenable.

- 1. **LOESS fit**

A LOESS fit was used as an exploratory analysis tool to identify trends in manual and automated segmentations (see **Figure S1**). Naïve rats have high ejection fractions (> 55%), while there is a higher degree of variability in the case of myocardial infarction rats whose EFs span a range from 30% up to 70%, approximately. The modelled EFs deviate from linearity in the extremes. For Op1, the diagonal switches sides at around the 50% EF mark. This trend is also identified for Op2, albeit it is less pronounced. No major differences are observed when comparing the fits between the two segmentation approaches.


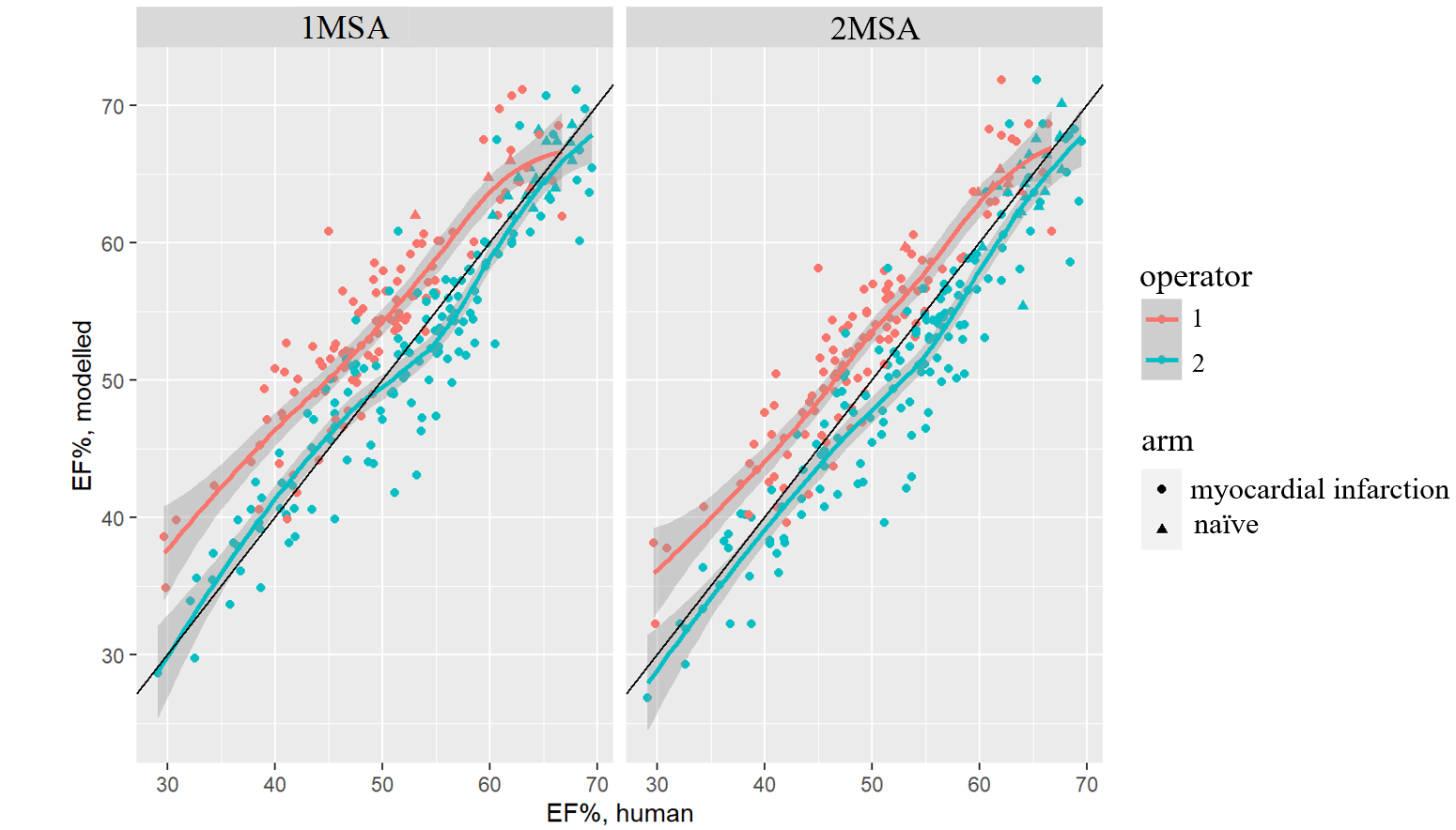


**Figure S1.** LOESS fit for EF determined using automated segmentation vs. manual segmentation by segmentation approach (left: one-model segmentation approach, right: two-model segmentation approach).

Both graphs plot the operators (─ Operator 1, ─ Operator 2) involved in the segmentation and the treatment arm (⦁ myocardial infarction, ▲ naïve)

1. **Implementation Details**
   1. **Linear Mixed Model**

The composite kernel consisting of a constant kernel multiplied by the radial-basis function (RBF) kernel is given by:

k$\text{(}\text{x}\text{,}\text{x'}\text{) = constant value }\text{∀}\text{ x, x'}$ Equation S2

*k*$\text{(}\text{x}\text{,}\text{x'}\text{) = exp}\left( \frac{\text{d}\left( \text{x}\text{,}\text{x'} \right)^{\text{2}}}{\text{2}\text{l}^{\text{2}}} \right)$ *Equation S3*

where the constant value for the constant kernel is set to 0.1 with constant value bounds between 0.1 and 10, *d*( · , · ) is the Euclidean distance, *l* is a length-scale parameter which is set to 0.5 with bounds 0.1 to 10.

1. **Model Selection Statistics**

**Table S1. DSC Statistic for Final Models in 2MSA^a^**

| model | phase model | Study 5 | Study 6 |
| --- | --- | --- | --- |
| Attention U-Net | systole | 0.90 ± 0.024 | 0.93 ± 0.019 |
|  | **diastole** | **0.95 ± 0.014** | **0.96 ± 0.011** |
| U-Net | systole | 0.90 ± 0.23 | 0.94 ± 0.017 |
| U-Net++ | diastole | 0.94 ± 0.013 | 0.90 ± 0.016 |
| V-Net | **systole** | **0.91 ± 0.022** | **0.94 ± 0.015** |
|  | diastole | 0.95 ± 0.013 | 0.95 ± 0.00087 |
| voting ensemble | systole | 0.91 ± 0.023 | 0.94 ± 0.015 |
|  | diastole | 0.95 ± 0.0095 | 0.86 ± 0.016 |
| averaging ensemble | systole | 0.89 ± 0.027 | 0.93 ± 0.023 |
|  | diastole | 0.95 ± 0.012 | 0.90 ± 0.015 |

***^a^*** DSC is reported as mean ± standard deviation

**Table S2.** **DSC Statistic for Final Models in 1MSA^a^**

| model | phase | Study 5 | Study 6 |
| --- | --- | --- | --- |
| Attention U-Net | systole | 0.90 ± 0.026 | 0.94 ± 0.019 |
|  | diastole | 0.95 ± 0.013 | 0.95 ± 0.014 |
| **U-Net** | **systole** | **0.90 ± 0.024** | **0.94 ± 0.018** |
|  | **diastole** | **0.95 ± 0.011** | **0.95 ± 0.014** |
| V-Net | systole | 0.90 ± 0.024 | 0.94 ± 0.025 |
|  | diastole | 0.95 ± 0.013 | 0.95 ± 0.021 |
| voting ensemble | systole | 0.90 ± 0.024 | 0.94 ± 0.019 |
|  | diastole | 0.96 ± 0.013 | 0.96 ± 0.0079 |
| averaging  ensemble | systole | 0.88 ± 0.030 | 0.94 ± 0.025 |
|  | diastole | 0.95 ± 0.015 | 0.96 ± 0.016 |

***^a^*** DSC is reported as mean ± standard deviation

The Hausdorff Distance (HD) from A to B is defined as follows:

$\tilde{\delta}_{H}\text{(A,B)}\text{ = }\max_{\text{a }\text{∈}\text{ A}} \min_{\text{b }\text{∈}\text{ B}} \left\| \text{a}\text{-}\text{b} \right\|$ *Equation S4*

and subsequently, the bidirectional Hausdorff distance is calculated as specified in *Equation 2*.

$\text{HD(A,B) = }\text{max(}{\tilde{\text{δ}}}_{\text{H}}\text{(A,B), }{\tilde{\text{δ}}}_{\text{H}}\text{(B,A)}\text{)}\text{ }$ Equation S5

Intraclass Correlation Coefficient (ICC) was determined to evaluate agreement between the actual volumes (derived from the true masks) and the predicted volumes. A value of 1 indicates perfect reliability, whereas a value of 0 indicates no agreement.

**Table S3. HD Statistic for Final Models in 2MSA^a^**

| model | phase model | Study 5 | Study 6 |
| --- | --- | --- | --- |
| Attention U-Net | systole | 2.0 ± 0.45 | 1.6 ± 0.31 |
|  | diastole | 1.63 ± 0.27 | 1.7 ± 1.1 |
| U-Net | systole | 2.0 ± 0.41 | 1.6 ± 0.27 |
| U-Net++ | diastole | 1.83 ± 0.50 | 2.0 ± 0.97 |
| V-Net | systole | 2.1 ± 0.44 | 1.59 ± 0.13 |
|  | diastole | 1.61 ± 0.28 | 1.8 ± 1.2 |
| voting ensemble | systole | 1.9 ± 0.40 | 1.52 ± 0.18 |
|  | diastole | 1.6 ± 0.17 | 2.12 ± 0.95 |
| averaging ensemble | systole | 2.1 ± 0.48 | 1.64 ± 0.23 |
|  | diastole | 1.79 ± 0.48 | 1.9 ± 0.98 |

***^a^*** HD is reported as mean ± standard deviation

**Table S4. ICC Statistic for Final Models in 2MSA^a^**

| model | phase model | Study 5 | Study 6 |
| --- | --- | --- | --- |
| Attention U-Net | systole | 0.79  (0.71 to 0.85) | 0.98  (0.96 to 0.99) |
|  | diastole | 0.90  (0.86 to 0.93) | 0.96  (0.92 to 0.98) |
| U-Net | systole | 0.79  (0.71 to 0.85) | 0.99  (0.97 to 0.99) |
| U-Net++ | diastole | 0.92  (0.88 to 0.94) | 0.59  (0.34 to 0.76) |
| V-Net | systole | 0.84  (0.78 to 0.89) | 0.99  (0.98 to 0.99) |
|  | diastole | 0.88  (0.84 to 0.92) | 0.92  (0.86 to 0.96) |
| voting ensemble | systole | 0.81  (0.73 to 0.86) | 0.99  (0.98 to 0.99) |
|  | diastole | 0.93  (0.90 to 0.95) | 0.30  (0.000 to 0.57) |
| averaging ensemble | systole | 0.66  (0.54 to 0.75) | 0.96  (0.93 to 0.98) |
|  | diastole | 0.92  (0.88 to 0.94) | 0.62  (0.38 to 0.78) |

***^a^*** ICC volume as mean and 95% confidence interval in brackets

**Table S5. HD Statistic for Final Models in 1MSA^a^**

| model | phase model | Study 5 | Study 6 |
| --- | --- | --- | --- |
| Attention U-Net | systole | 2.0 ± 0.38 | 1.7 ± 0.52 |
|  | diastole | 1.6 ± 0.32 | 1.7 ± 1.2 |
| U-Net | systole | 2.0 ± 0.48 | 1.6 ± 0.20 |
|  | diastole | 1.6 ± 0.26 | 1.8 ± 1.2 |
| V-Net | systole | 2.1 ± 0.47 | 1.6 ± 0.20 |
|  | diastole | 1.6 ± 0.31 | 1.8 ± 1.2 |
| voting ensemble | systole | 2.0 ± 0.46 | 1.6 ± 0.17 |
|  | diastole | 1.6 ± 0.28 | 1.7 ± 1.2 |
| averaging ensemble | systole | 2.2 ± 0.49 | 1.7 ± 0.23 |
|  | diastole | 1.7 ± 0.33 | 1.7 ± 1.2 |

***^a^*** HD is reported as mean ± standard deviation

**Table S6. ICC Statistic for Final Models in 1MSA^a^**

| model | phase model | Study 5 | Study 6 |
| --- | --- | --- | --- |
| Attention U-Net | systole | 0.79  (0.70 to 0.84) | 0.98  (0.97 to 0.99) |
|  | diastole | 0.91  (0.87 to 0.94) | 0.95  (0.90 to 0.97) |
| U-Net | systole | 0.80  (0.72 to 0.85) | 0.98  (0.95 to 0.99) |
|  | diastole | 0.91  (0.88 to 0.94) | 0.94  (0.88 to 0.97) |
| V-Net | systole | 0.79  (0.71 to 0.85) | 0.97  (0.95 to 0.99) |
|  | diastole | 0.94  (0.91 to 0.96) | 0.95  (0.91 to 0.97) |
| voting ensemble | systole | 0.79  (0.71 to 0.85) | 0.98  (0.96 to 0.99) |
|  | diastole | 0.92  (0.88 to 0.94) | 0.95  (0.90 to 0.97) |
| averaging ensemble | systole | 0.62  (0.49 to 0.72) | 0.95  (0.91 to 0.97) |
|  | diastole | 0.81  (0.74 to 0.86) | 0.88  (0.78 to 0.93) |

***^a^*** ICC volume as mean and 95% confidence interval in brackets


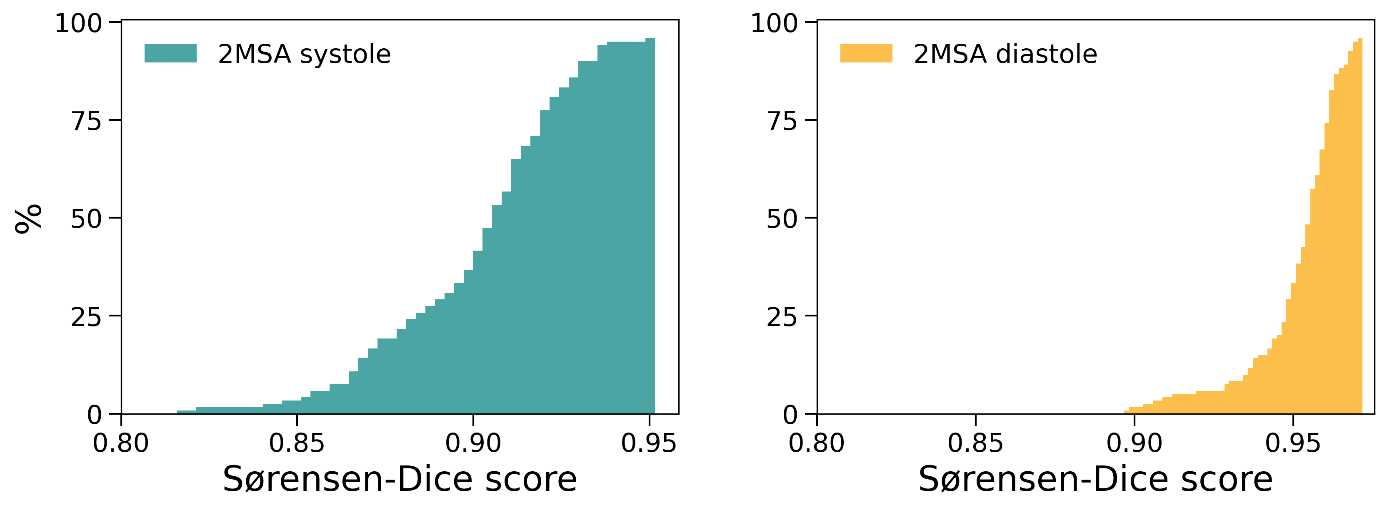


**B**

**A**

**Figure S2.** Sørensen-Dice score cumulative distribution for 2MSA.

**A**: V-Net

**B**: Attention U-Net


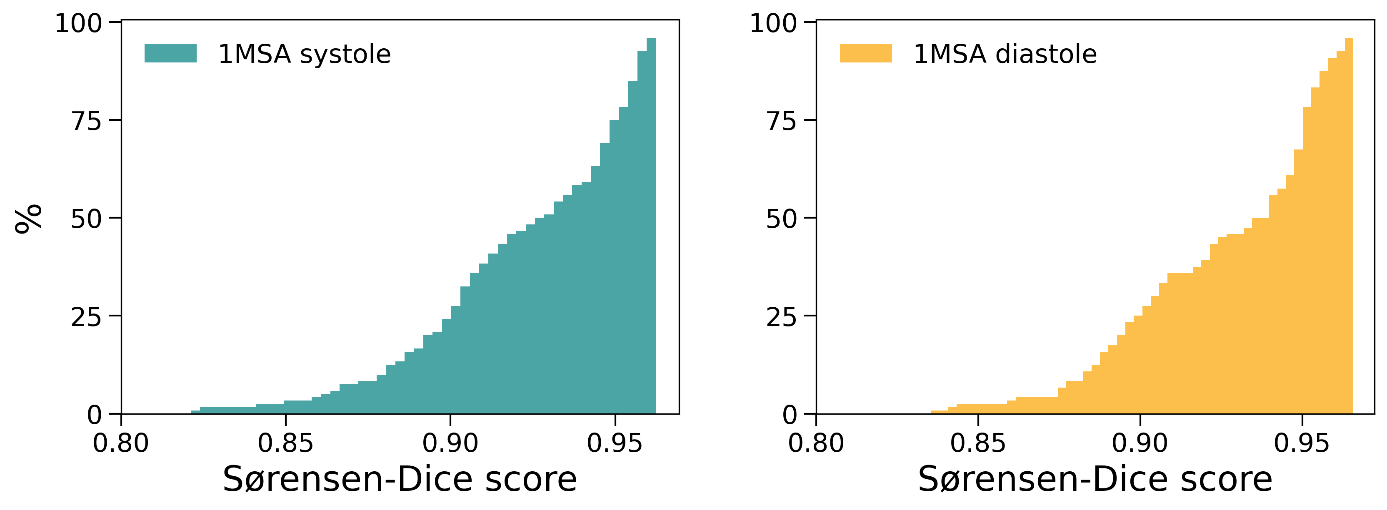


**Figure S3.** Sørensen-Dice score cumulative distribution for 1MSA U-Net model

1. **Segmentation Quality Analysis by Slice**


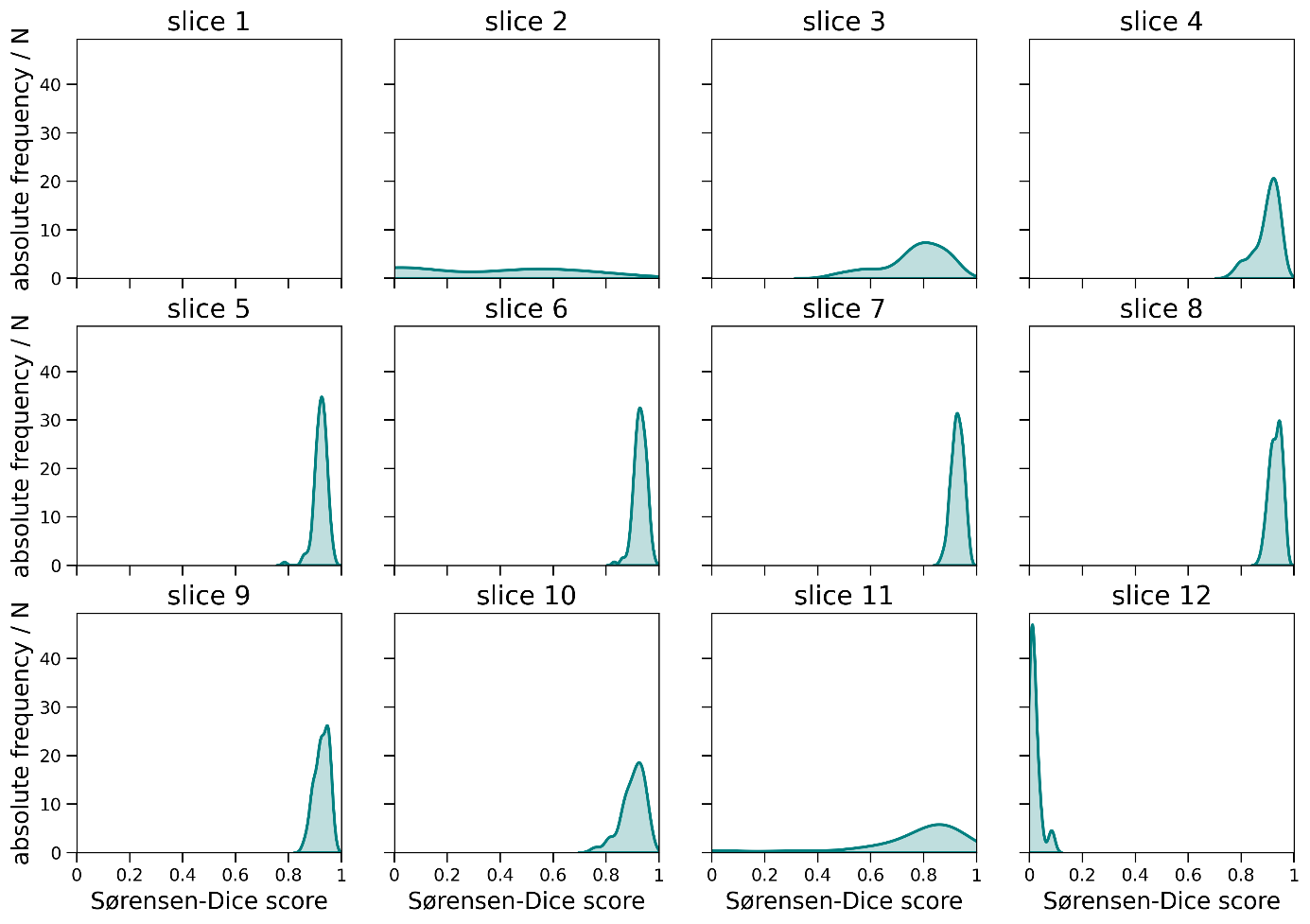


**Figure S4.** Sørensen-Dice score distribution per slice for systolic phase segmentations, where the slice 1 is the basal slice and the last slice the apical slice.


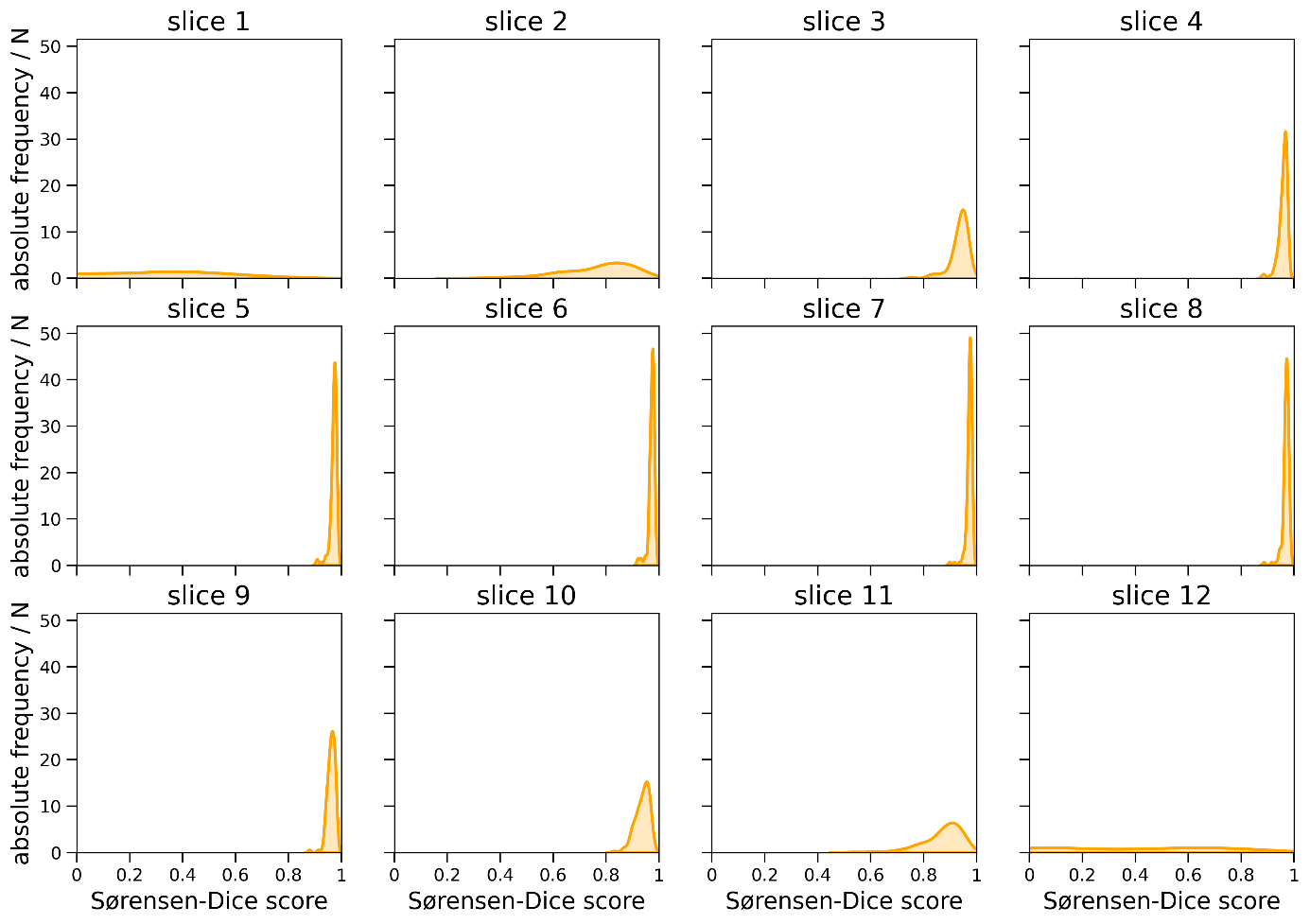


**Figure S5**. Sørensen-Dice score distribution per slice for diastolic phase segmentations, where the slice 1 is the basal slice and the last slice the apical slice.

1. **Noise Robustness Analysis**

**Table S7. Noise Robustness Analysis for Two-model Segmentation Approach**

| approach | architecture phase | metric | model type | Gaussian noise | Rician noise | Rayleigh noise | mixed noise |
| --- | --- | --- | --- | --- | --- | --- | --- |
| two-model segmentation approach | Attention U-Net diastole | HD | non-augmented | 5.8 ± 3.9 | 6.0 ± 3.8 | 8.9 ± 3.4 | 12.0 ± 7.4 |
|  |  |  | augmented | 1.6 ± 0.27 | 1.6 ± 0.28 | 1.6 ± 0.27 | 1.7 ± 0.41 |
|  |  | ICC | non-augmented | 0.75  (0.66 to 0.82) | 0.72  (0.62 to 0.80) | 0.27  (0.093 to 0.43) | 0.26  (0.086 to 0.42) |
|  |  |  | augmented | 0.90  (0.86 to 0.94) | 0.89  (0.84 to 0.92) | 0.88  (0.84 to 0.92) | 0.83  (0.77 to 0.88) |
|  | V-Net  systole | HD | non-augmented | 2.1 ± 0.44 | 2.1 ± 0.46 | 2.1 ± 0.45 | 2.2 ± 0.47 |
|  |  |  | augmented | 1.9 ± 0.35 | 1.9 ± 0.35 | 2.0 ± 0.34 | 2.0 ± 0.40 |
|  |  | ICC | non-augmented | 0.70  (0.60 to 0.78) | 0.81  (0.74 to 0.87) | 0.80  (0.72 to 0.86) | 0.74  (0.64 to 0.81) |
|  |  |  | augmented | 0.84  (0.78 to 0.89) | 0.83  (0.77 to 0.88) | 0.82  (0.76 to 0.88) | 0.80  (0.72 to 0.85) |

**Table S8. Noise Robustness Analysis for One-model Segmentation Approach**

| approach | architecture | phase | metric | model type | Gaussian noise | Rician noise | Rayleigh noise | mixed noise |
| --- | --- | --- | --- | --- | --- | --- | --- | --- |
| one-model segmentation approach | U-Net | diastole | HD | non-augmented | 1.7 ± 0.30 | 1.7 ± 0.31 | 1.6 ± 0.27 | 1.8 ± 0.50 |
|  |  |  |  | augmented | 1.8 ± 0.42 | 2.0 ± 0.50 | 1.6 ± 0.29 | 2.1 ± 0.54 |
|  |  |  | ICC | non-augmented | 0.88  (0.84 to 0.92) | 0.88  (0.83 to 0.91) | 0.91  (0.87 to 0.93) | 0.88  (0.84 to 0.92) |
|  |  |  |  | augmented | 0.91  (0.87 to 0.94) | 0.90  (0.86 to 0.93) | 0.90  (0.85 to 0.0.927) | 0.87  (0.82 to 0.91) |
|  |  | systole | HD | non-augmented | 2.2 ± 0.49 | 2.2 ± 0.48 | 2.2 ± 0.48 | 2.8 ± 1.6 |
|  |  |  |  | augmented | 1.9 ± 0.44 | 1.6 ± 0.26 | 2.0 ± 0.51 | 1.6 ± 0.30 |
|  |  |  | ICC | non-augmented | 0.79  (0.71 to 0.85) | 0.79  (0.71 to 0.85) | 0.76  (0.67 to 0.83) | 0.73  (0.64 to 0.81) |
|  |  |  |  | augmented | 0.77  (0.69 to 0.84) | 0.77  (0.68 to 0.83) | 0.76  (0.68 to 0.83) | 0.70  (0.59 to 0.78) |

1. **Hyperparameter Tuning**

**Table S9. Blocks, layers and downsampling dimensions hyperparameter tuning for systole model (two-model segmentation approach) ^a^**

| **architecture** | **blocks** | **layers** | **downsampling dimensions** | **DSC** |
| --- | --- | --- | --- | --- |
| Attention U-Net | 2 | 4 | 3 | 0.91449 |
| Attention U-Net | 2 | 2 | 2 | 0.90946 |
| Attention U-Net | 4 | 4 | 3 | 0.90003 |
| Attention U-Net | 3 | 4 | 2 | 0.9102 |
| Attention U-Net | 4 | 6 | 3 | 0.89254 |
| Attention U-Net | 2 | 4 | 3 | 0.91517 |
| Attention U-Net | 3 | 8 | 2 | 0.90992 |
| Attention U-Net | 2 | 8 | 3 | 0.91959 |
| **Attention U-Net** | **3** | **2** | **3** | **0.92306** |
| Attention U-Net | 3 | 2 | 2 | 0.91162 |
| U-Net | 3 | 2 | 2 | 0.91095 |
| U-Net | 5 | 8 | 3 | 0.92392 |
| U-Net | 4 | 4 | 2 | 0.91780 |
| U-Net | 4 | 6 | 3 | 0.69443 |
| U-Net | 3 | 4 | 3 | 0.91719 |
| U-Net | 2 | 2 | 3 | 0.219913 |
| U-Net | 2 | 4 | 3 | 0.50917 |
| **U-Net** | **5** | **2** | **2** | **0.91466** |
| U-Net | 2 | 8 | 3 | 0.91249 |
| U-Net | 4 | 8 | 2 | 0.90594 |
| V-Net | 2 | 2 | 3 | 0.91436 |
| V-Net | 2 | 6 | 2 | 0.92544 |
| V-Net | 3 | 2 | 2 | 0.92006 |
| V-Net | 4 | 4 | 3 | 0.9205 |
| V-Net | 3 | 6 | 3 | 0.91475 |
| **V-Net** | **4** | **6** | **2** | **0.92596** |
| V-Net | 2 | 8 | 3 | 0.86622 |
| V-Net | 4 | 8 | 2 | 0.91496 |
| V-Net | 3 | 8 | 2 | 0.90558 |
| V-Net | 4 | 2 | 2 | 0.92054 |

**^a^** The model with the highest DSC in each of the architectures tried is highlighted in bold

**Table S10. Hyperparameter tuning for systole model (two-model segmentation approach) ^a^**

| **architecture**  **(blocks, layers, dimensions)** | **kernel size** | **number of filters** | **renormalisation** | **activation function** | **dropout** | **batch size** | **optimiser** | **learnrate** | **pooling** | **kernel initialiser** | **deconvolution** | **DSC** |
| --- | --- | --- | --- | --- | --- | --- | --- | --- | --- | --- | --- | --- |
| Attention U-Net (3,2,3) | (5, 5, 5) | 32 | True | ELU | 0.2 | 32 | Adam | 10^-3^ | average | random normal | False | 0.91305 |
| Attention U-Net (3,2,3) | (2, 2, 2) | 8 | True | SELU | 0.4 | 16 | Adam | 10^-3^ | average | random normal | False | 0.63149 |
| Attention U-Net (3,2,3) | (3, 3, 3) | 16 | True | SELU | 0 | 64 | Adam | 10^-3^ | max | Glorot uniform | True | 0.87994 |
| Attention U-Net (3,2,3) | (5, 5, 5) | 32 | False | GELU | 0.4 | 32 | Adam | 10^-3^ | average | Glorot uniform | True | 0.07667 |
| Attention U-Net (3,2,3) | (5, 5, 5) | 16 | True | SELU | 0.2 | 32 | Adam | 10^-2^ | max | Glorot uniform | True | 0.91216 |
| Attention U-Net (3,2,3) | (2, 2, 2) | 8 | True | GELU | 0.2 | 64 | Adam | 10^-3^ | average | Glorot normal | False | 0.6799 |
| Attention U-Net (3,2,3) | (3, 3, 3) | 16 | False | GELU | 0.6 | 16 | RMSProp | 10^-2^ | average | Glorot normal | False | 0.08205 |
| Attention U-Net (3,2,3) | (5, 5, 5) | 16 | False | SELU | 0.4 | 16 | RMSProp | 10^-3^ | average | Glorot normal | True | 0.40656 |
| Attention U-Net (3,2,3) | (2, 2, 2) | 16 | True | GELU | 0.2 | 32 | Adam | 10^-2^ | max | glorot normal | False | 0.90011 |
| Attention U-Net (3,2,3) | (2, 2, 2) | 32 | False | ELU | 0.6 | 32 | Adam | 10^-2^ | max | glorot normal | False | 0.59155 |
| Attention U-Net (3,2,3) | (2, 2, 2) | 32 | True | SELU | 0.4 | 16 | Adam | 10^-3^ | max | Glorot normal | True | 0.77934 |
| Attention U-Net (3,2,3) | (3, 3, 3) | 8 | False | GELU | 0.4 | 64 | RMSProp | 10^-2^ | max | Glorot normal | False | 0.08081 |

*(continuation)*

| **architecture**  **(blocks, layers, dimensions)** | **kernel size** | **number of filters** | **renormalisation** | **activation function** | **dropout** | **batch size** | **optimiser** | **learnrate** | **pooling** | **kernel initialiser** | **deconvolution** | **DSC** |
| --- | --- | --- | --- | --- | --- | --- | --- | --- | --- | --- | --- | --- |
| Attention U-Net (3,2,3) | (5, 5, 5) | 8 | True | swish | 0.0 | 8 | Adam | 10^-2^ | average | Glorot normal | False | 0.92118 |
| Attention U-Net (3,2,3) | (5, 5, 5) | 8 | True | GELU | 0.6 | 32 | Adam | 10^-2^ | max | random normal | False | 0.64864 |
| Attention U-Net (3,2,3) | (5, 5, 5) | 16 | False | GELU | 0.4 | 32 | Adam | 10^-2^ | average | Glorot normal | False | 0.10512 |
| Attention U-Net (3,2,3) | (3, 3, 3) | 32 | True | ELU | 0.2 | 16 | RMSProp | 10^-2^ | max | Glorot normal | False | 0.79341 |
| Attention U-Net (3,2,3) | (3, 3, 3) | 8 | True | ELU | 0.2 | 16 | Adam | 10^-3^ | max | Glorot uniform | True | 0.75997 |
| Attention U-Net (3,2,3) | (5, 5, 5) | 8 | False | GELU | 0.6 | 8 | Adam | 10^-3^ | max | random normal | False | 0.01196 |
| Attention U-Net (3,2,3) | (3, 3, 3) | 16 | True | swish | 0.6 | 64 | Adam | 10^-2^ | average | Glorot normal | False | 0.27207 |
| Attention U-Net (3,2,3) | (3, 3, 3) | 16 | True | ELU | 0 | 8 | Adam | 10^-2^ | max | Glorot normal | True | 0.92306 |
| Attention U-Net (3,2,3) | (3, 3, 3) | 16 | True | ELU | 0 | 64 | Adam | 10^-2^ | max | Glorot normal | True | 0.89907 |
| Attention U-Net (3,2,3) | (3, 3, 3) | 16 | True | ELU | 0.2 | 8 | Adam | 10^-2^ | max | Glorot normal | True | 0.8925 |
| Attention U-Net (3,2,3) | (3, 3, 3) | 16 | True | swish | 0 | 8 | Adam | 10^-2^ | max | Glorot normal | True | 0.33729 |
| Attention U-Net (3,2,3) | (3, 3, 3) | 16 | True | ELU | 0 | 8 | Adam | 10^-2^ | max | Glorot normal | False | 0.89186 |
| Attention U-Net (3,2,3) | (3, 3, 3) | 16 | True | GELU | 0 | 8 | Adam | 10^-2^ | max | Glorot normal | True | 0.89368 |
| U-Net  (5,2,2) | (5, 5, 5) | 8 | True | swish | 0 | 8 | Adam | 10^-2^ | max | Glorot normal | False | 0.89443 |
| U-Net  (5,2,2) | (5, 5, 5) | 8 | True | swish | 0 | 32 | Adam | 10^-2^ | average | Glorot normal | False | 0.89288 |
| U-Net  (5,2,2) | (5, 5, 5) | 8 | True | swish | 0 | 8 | Adam | 10^-2^ | average | Glorot normal | True | 0.89546 |

*(continuation)*

| **architecture**  **(blocks, layers, dimensions)** | **kernel size** | **number of filters** | **renormalisation** | **activation function** | **dropout** | **batch size** | **optimiser** | **learnrate** | **pooling** | **kernel initialiser** | **deconvolution** | **DSC** |
| --- | --- | --- | --- | --- | --- | --- | --- | --- | --- | --- | --- | --- |
| U-Net  (5,2,2) | (5, 5, 5) | 8 | True | GELU | 0 | 8 | Adam | 10^-2^ | average | Glorot normal | False | 0.89465 |
| U-Net  (5,2,2) | (3, 3, 3) | 16 | True | ELU | 0 | 8 | Adam | 10^-2^ | max | Glorot normal | True | 0.92306 |
| U-Net  (5,2,2) | (3, 3, 3) | 16 | False | SELU | 0 | 16 | RMSProp | 10^-3^ | max | Glorot normal | True | 0.28012 |
| U-Net  (5,2,2) | (3, 3, 3) | 16 | True | SELU | 0.2 | 8 | Adam | 10^-3^ | max | Glorot uniform | True | 0.90423 |
| U-Net  (5,2,2) | (2, 2, 2) | 8 | True | ELU | 0 | 16 | Adam | 10^-2^ | average | random normal | True | 0.90533 |
| U-Net  (5,2,2) | (3, 3, 3) | 16 | False | swish | 0.2 | 8 | Adam | 10^-2^ | max | Glorot uniform | False | 0.93156 |
| U-Net  (5,2,2) | (5, 5, 5) | 8 | True | GELU | 0.2 | 32 | Adam | 10^-2^ | average | random normal | False | 0.01196 |
| U-Net  (5,2,2) | (3, 3, 3) | 16 | False | ELU | 0.4 | 32 | RMSProp | 10^-2^ | max | Glorot uniform | True | 0.48532 |
| U-Net  (5,2,2) | (5, 5, 5) | 32 | True | swish | 0.6 | 8 | RMSProp | 10^-2^ | average | Glorot uniform | True | 0.08121 |
| U-Net  (5,2,2) | (5, 5, 5) | 8 | True | GELU | 0.4 | 16 | Adam | 10^-2^ | average | Glorot uniform | False | 0.92159 |
| U-Net  (5,2,2) | (3, 3, 3) | 16 | True | SELU | 0.2 | 64 | RMSProp | 10^-3^ | max | Glorot normal | True | 0.16392 |
| U-Net  (5,2,2) | (3, 3, 3) | 16 | False | SELU | 0.6 | 64 | RMSProp | 10^-3^ | max | Glorot uniform | True | 0.29401 |
| U-Net  (5,2,2) | (5, 5, 5) | 32 | True | GELU | 0.2 | 16 | RMSProp | 10^-2^ | max | Glorot uniform | True | 0.88553 |
| U-Net  (5,2,2) | (2, 2, 2) | 8 | False | ELU | 0 | 32 | Adam | 10^-3^ | average | Glorot normal | False | 0.53106 |
| U-Net  (5,2,2) | (5, 5, 5) | 16 | False | ELU | 0.2 | 8 | RMSProp | 10^-3^ | max | Glorot uniform | False | 0.6165 |
| U-Net  (5,2,2) | (3, 3, 3) | 16 | False | GELU | 0.2 | 8 | Adam | 10^-2^ | max | Glorot uniform | False | 0.65958 |
| U-Net  (5,2,2) | (3, 3, 3) | 16 | True | swish | 0.2 | 8 | Adam | 10^-2^ | max | Glorot uniform | False | 0.9153 |
| U-Net  (5,2,2) | (3, 3, 3) | 16 | False | swish | 0 | 8 | Adam | 10^-2^ | max | Glorot uniform | False | 0.07589 |
| V-Net  (4,6,2) | (2, 2, 2) | 16 | True | GELU | 0.6 | 32 | Adam | 10^-3^ | max | random normal | True | 0.4701 |
| V-Net  (4,6,2) | (3, 3, 3) | 32 | True | GELU | 0.6 | 16 | RMSProp | 10^-3^ | average | Glorot normal | True | 0.07824 |
| V-Net  (4,6,2) | (2, 2, 2) | 8 | False | GELU | 0.4 | 8 | RMSProp | 10^-2^ | average | random normal | True | 0.07858 |
| V-Net  (4,6,2) | (3, 3, 3) | 8 | False | ELU | 0.4 | 32 | Adam | 10^-3^ | max | Glorot uniform | True | 0.17348 |
| V-Net  (4,6,2) | (2, 2, 2) | 16 | True | GELU | 0.2 | 32 | Adam | 10^-2^ | max | Glorot normal | True | 0.90206 |
| V-Net  (4,6,2) | (2, 2, 2) | 16 | True | SELU | 0.6 | 32 | RMSProp | 10^-3^ | average | random normal | True | 0.0779 |
| V-Net  (4,6,2) | (2, 2, 2) | 32 | True | swish | 0.2 | 32 | Adam | 10^-3^ | max | Glorot normal | True | 0.86483 |
| V-Net  (4,6,2) | (2, 2, 2) | 8 | True | ELU | 0.4 | 16 | Adam | 10^-3^ | average | Glorot normal | True | 0.36272 |

*(continuation)*

| **architecture**  **(blocks, layers, dimensions)** | **kernel size** | **number of filters** | **renormalisation** | **activation function** | **dropout** | **batch size** | **optimiser** | **learnrate** | **pooling** | **kernel initialiser** | **deconvolution** | **DSC** |
| --- | --- | --- | --- | --- | --- | --- | --- | --- | --- | --- | --- | --- |
| V-Net  (4,6,2) | (3, 3, 3) | 16 | True | ELU | 0 | 8 | Adam | 10^-2^ | max | Glorot normal | True | 0.92596 |
| V-Net  (4,6,2) | (2, 2, 2) | 32 | True | ELU | 0.2 | 8 | Adam | 10^-3^ | max | random normal | True | 0.84278 |
| V-Net  (4,6,2) | (2, 2, 2) | 32 | True | swish | 0.4 | 32 | RMSProp | 10^-3^ | max | random normal | True | 0.07769 |

**^a^** The model with the highest DSC in each of the architectures tried is highlighted in bold

**Table S11. Blocks, layers and downsampling dimensions hyperparameter tuning for diastole model (two-model segmentation approach) ^a^**

**^a^** The model with the highest DSC in each of the architectures tried is highlighted in bold

| **architecture** | **blocks** | **layers** | **dimensions** | **DSC** |
| --- | --- | --- | --- | --- |
| U-Net++ | 4 | 8 | 3 | 0.92874 |
| U-Net++ | 3 | 4 | 3 | 0.944933333 |
| U-Net++ | 4 | 4 | 2 | 0.920933333 |
| U-Net++ | 3 | 6 | 3 | 0.601446667 |
| **U-Net++** | **3** | **8** | **2** | **0.952003333** |
| U-Net++ | 4 | 6 | 2 | 0.926186667 |
| Attention U-Net | 4 | 4 | 2 | 0.95433 |
| Attention U-Net | 4 | 4 | 3 | 0.95056 |
| Attention U-Net | 4 | 6 | 2 | 0.95357 |
| Attention U-Net | 2 | 4 | 3 | 0.94697 |
| **Attention U-Net** | **4** | **6** | **3** | **0.95544** |
| Attention U-Net | 2 | 8 | 3 | 0.94434 |
| Attention U-Net | 3 | 6 | 2 | 0.95193 |
| Attention U-Net | 3 | 4 | 2 | 0.95018 |
| Attention U-Net | 4 | 2 | 2 | 0.95056 |
| Attention U-Net | 2 | 8 | 3 | 0.86902 |
| V-Net | 2 | 4 | 3 | 0.94244 |
| V-Net | 2 | 6 | 3 | 0.95183 |
| V-Net | 2 | 8 | 3 | 0.94686 |
| V-Net | 2 | 8 | 2 | 0.95652 |
| V-Net | 4 | 8 | 3 | 0.95042 |
| V-Net | 3 | 2 | 3 | 0.94937 |
| **V-Net** | **3** | **4** | **2** | **0.95797** |
| V-Net | 3 | 6 | 3 | 0.95287 |
| V-Net | 2 | 2 | 3 | 0.93696 |
| V-Net | 4 | 6 | 3 | 0.95145 |

**Table S12. Hyperparameter tuning for diastole model (two-model segmentation approach)^a^**

| **architecture**  **(blocks, layers, dimensions)** | **kernel size** | **number of filters** | **renormalisation** | **activation function** | **dropout** | **batch size** | **optimiser** | **Learning rate** | **pooling** | **kernel initialiser** | **deconvolution** | **DSC** |
| --- | --- | --- | --- | --- | --- | --- | --- | --- | --- | --- | --- | --- |
| U-Net++  (3,8,2) | (2, 2, 2) | 32 | FALSE | SELU | 0.4 | 16 | Adam | 10^-2^ | max | Glorot normal | TRUE | 0.558886667 |
| U-Net++  (3,8,2) | (3, 3, 3) | 8 | TRUE | GELU | 0.6 | 16 | Adam | 10^-2^ | max | Glorot uniform | FALSE | 0.53002 |
| U-Net++  (3,8,2) | (5, 5, 5) | 8 | FALSE | GELU | 0.6 | 32 | RMSProp | 10^-2^ | max | Glorot normal | FALSE | 0.594326667 |
| U-Net++  (3,8,2) | (3, 3, 3) | 16 | FALSE | swish | 0.2 | 8 | Adam | 10^-2^ | max | Glorot uniform | FALSE | 0.67312 |
| U-Net++  (3,8,2) | (3, 3, 3) | 32 | TRUE | ELU | 0.2 | 32 | Adam | 10^-3^ | average | random normal | TRUE | 0.919523333 |
| U-Net++  (3,8,2) | (5, 5, 5) | 8 | TRUE | GELU | 0.2 | 16 | RMSProp | 10^-2^ | max | Glorot normal | FALSE | 0.87706 |
| U-Net++  (3,8,2) | (3, 3, 3) | 8 | TRUE | GELU | 0.4 | 32 | RMSProp | 10^-2^ | average | random normal | TRUE | 0.61959 |
| U-Net++  (3,8,2) | (5, 5, 5) | 16 | FALSE | GELU | 0.2 | 16 | Adam | 10^-3^ | average | random normal | FALSE | 0.714096667 |
| U-Net++  (3,8,2) | (5, 5, 5) | 32 | FALSE | SELU | 0.6 | 8 | Adam | 10^-3^ | average | random normal | FALSE | 0.76115 |
| U-Net++  (3,8,2) | (3, 3, 3) | 16 | TRUE | SELU | 0.4 | 32 | Adam | 10^-2^ | max | Glorot normal | FALSE | 0.817603333 |
| **U-Net++**  **(3,8,2)** | **(3, 3, 3)** | **16** | **TRUE** | **ELU** | **0** | **8** | **Adam** | **10^-3^** | **max** | **Glorot normal** | **TRUE** | **0.952003333** |
| U-Net++  (3,8,2) | (3, 3, 3) | 16 | TRUE | swish | 0 | 8 | Adam | 10^-3^ | max | Glorot normal | TRUE | 0.89604 |
| U-Net++  (3,8,2) | (5, 5, 5 | 16 | TRUE | ELU | 0.2 | 8 | Adam | 10^-3^ | max | Glorot normal | TRUE | 0.65265 |
| U-Net++  (3,8,2) | (3, 3, 3) | 16 | TRUE | ELU | 0 | 8 | Adam | 10^-3^ | average | Glorot normal | TRUE | 0.25532 |
| U-Net++  (3,8,2) | (3, 3, 3) | 16 | TRUE | ELU | 0 | 8 | Adam | 10^-3^ | max | Glorot normal | FALSE | 0.32035 |
| Attention U-Net  (4,6,3) | (2, 2, 2) | 32 | TRUE | swish | 0.2 | 16 | Adam | 10^-3^ | average | Glorot normal | FALSE | 0.7399 |

*(continuation)*

| **architecture**  **(blocks, layers, dimensions)** | **kernel size** | **number of filters** | **renormalisation** | **activation function** | **dropout** | **batch size** | **optimiser** | **learning rate** | **pooling** | **kernel initialiser** | **deconvolution** | **DSC** |
| --- | --- | --- | --- | --- | --- | --- | --- | --- | --- | --- | --- | --- |
| Attention U-Net  (4,6,3) | (2, 2, 2) | 8 | TRUE | ELU | 0 | 8 | RMSProp | 10^-2^ | average | Glorot uniform | TRUE | 0.72102 |
| Attention U-Net  (4,6,3) | (5, 5, 5) | 16 | TRUE | swish | 0.6 | 32 | RMSProp | 10^-3^ | max | Glorot uniform | TRUE | 0.14878 |
| Attention U-Net  (4,6,3) | (5, 5, 5) | 16 | FALSE | ELU | 0.4 | 32 | RMSProp | 10^-3^ | average | Glorot uniform | FALSE | 0.14967 |
| Attention U-Net  (4,6,3) | (3, 3, 3) | 32 | FALSE | ELU | 0.6 | 16 | RMSProp | 10^-2^ | max | Glorot uniform | FALSE | 0.1461 |
| Attention U-Net  (4,6,3) | (3, 3, 3) | 16 | TRUE | ELU | 0 | 8 | Adam | 10^-3^ | max | Glorot normal | TRUE | 0.95140 |
| Attention U-Net  (4,6,3) | (3, 3, 3) | 16 | TRUE | swish | 0 | 8 | Adam | 10^-3^ | max | Glorot normal | TRUE | 0.94496 |
| **Attention U-Net**  **(4,6,3)** | **(3, 3, 3)** | **16** | **TRUE** | **ELU** | **0** | **8** | **Adam** | **10^-3^** | **average** | **Glorot normal** | **TRUE** | **0.95198** |
| Attention U-Net  (4,6,3) | (3, 3, 3) | 16 | TRUE | ELU | 0 | 8 | Adam | 10^-3^ | max | Glorot normal | FALSE | 0.95007 |
| V-Net  (3,4,2) | (5, 5, 5) | 16 | TRUE | ELU | 0.4 | 16 | Adam | 10^-3^ | average | Glorot normal | TRUE | 0.9379 |
| **V-Net**  **(3,4,2)** | **(3, 3, 3)** | **16** | **TRUE** | **ELU** | **0** | **8** | **Adam** | **10^-3^** | **max** | **Glorot normal** | **TRUE** | **0.958** |
| V-Net  (3,4,2) | (5, 5, 5) | 8 | FALSE | ELU | 0.4 | 16 | Adam | 10^-3^ | average | Glorot normal | TRUE | 0.93235 |
| V-Net  (3,4,2) | (3, 3, 3) | 32 | TRUE | GELU | 0.4 | 64 | Adam | 10^-3^ | max | Glorot uniform | TRUE | 0.19961 |
| V-Net  (3,4,2) | (2, 2, 2) | 8 | FALSE | SELU | 0.4 | 32 | Adam | 10^-3^ | max | random normal | TRUE | 0.44774 |
| V-Net  (3,4,2) | (5, 5, 5) | 16 | FALSE | ELU | 0.6 | 64 | Adam | 10^-3^ | max | random normal | TRUE | 0.17005 |
| V-Net  (3,4,2) | (2, 2, 2) | 16 | TRUE | SELU | 0.4 | 64 | Adam | 10^-3^ | average | random normal | TRUE | 0.82687 |
| V-Net  (3,4,2) | (5, 5, 5) | 8 | FALSE | swish | 0.6 | 16 | Adam | 10^-2^ | max | random normal | TRUE | 0.89169 |
| V-Net  (3,4,2) | (5, 5, 5) | 8 | TRUE | SELU | 0 | 8 | Adam | 10^-3^ | max | Glorot uniform | TRUE | 0.92336 |
| V-Net  (3,4,2) | (5, 5, 5) | 32 | FALSE | swish | 0.2 | 32 | Adam | 10^-2^ | max | Glorot normal | TRUE | 0.39526 |
| V-Net  (3,4,2) | (5, 5, 5) | 16 | TRUE | swish | 0.4 | 32 | Adam | 10^-2^ | average | Glorot normal | TRUE | 0.93785 |
| V-Net  (3,4,2) | (5, 5, 5) | 8 | TRUE | SELU | 0.4 | 32 | Adam | 10^-3^ | max | random normal | TRUE | 0.81267 |

**^a^** The model with the highest DSC in each of the architectures tried is highlighted in bold

**Table S13. Blocks, layers and downsampling dimensions hyperparameter tuning for diastole model (one-model segmentation approach)** **^a^**

| **Architecture** | **Blocks** | **Layers** | **Dimensions** | **DSC** |
| --- | --- | --- | --- | --- |
| Attention U-Net | 3 | 6 | 3 | 0.86829 |
| Attention U-Net | 2 | 2 | 2 | 0.88033 |
| Attention U-Net | 3 | 8 | 2 | 0.89134 |
| Attention U-Net | 2 | 8 | 2 | 0.90954 |
| Attention U-Net | 2 | 8 | 3 | 0.62085 |
| Attention U-Net | 3 | 2 | 3 | 0.935 |
| Attention U-Net | 3 | 2 | 2 | 0.92557 |
| Attention U-Net | 2 | 8 | 2 | 0.76726 |
| Attention U-Net | 3 | 4 | 2 | 0.92665 |
| V-Net | 4 | 4 | 2 | 0.91871 |
| V-Net | 4 | 2 | 2 | 0.92691 |
| V-Net | 2 | 2 | 2 | 0.92505 |
| V-Net | 3 | 8 | 3 | 0.90504 |
| V-Net | 4 | 8 | 3 | 0.91871 |
| V-Net | 2 | 4 | 2 | 0.88028 |
| V-Net | 4 | 2 | 3 | 0.94203 |
| V-Net | 3 | 2 | 3 | 0.94502 |
| V-Net | 2 | 8 | 3 | 0.9277 |
| U-Net | 4 | 6 | 3 | 0.92226 |
| U-Net | 4 | 4 | 2 | 0.82725 |
| U-Net | 4 | 4 | 2 | 0.9228 |
| U-Net | 2 | 4 | 2 | 0.93748 |
| U-Net | 3 | 8 | 2 | 0.92093 |
| U-Net | 3 | 8 | 2 | 0.93273 |
| U-Net | 3 | 4 | 2 | 0.94318 |
| U-Net | 3 | 6 | 2 | 0.90307 |
| U-Net++ | 2 | 2 | 3 | 0.969726667 |
| U-Net++ | 4 | 4 | 3 | 0.88282 |
| U-Net++ | 3 | 6 | 3 | 0.907096667 |
| U-Net++ | 2 | 4 | 3 | 0.97156 |
| U-Net++ | 4 | 6 | 2 | 0.536096667 |
| U-Net++ | 2 | 2 | 3 | 0.96086 |
| U-Net++ | 3 | 2 | 2 | 0.940423333 |
| U-Net++ | 3 | 6 | 2 | 0.954563333 |
| U-Net++ | 2 | 8 | 2 | 0.974 |
| U-Net++ | 4 | 8 | 2 | 0.92777 |

**^a^** The model with the highest DSC in each of the architectures tried is highlighted in bold

**Table S14. Hyperparameter tuning for model (one-model segmentation approach)^a^**

| **architecture**  **(blocks, layers, dimensions)** | **kernel size** | **number of filters** | **renormalisation** | **activation function** | **dropout** | **batch size** | **optimiser** | **learnrate** | **pooling** | **kernel initialiser** | **deconvolution** | **DSC** |
| --- | --- | --- | --- | --- | --- | --- | --- | --- | --- | --- | --- | --- |
| U-Net++  (2,8,2) | (5, 5, 5) | 16 | True | ELU | 0.2 | 32 | Adam | 10^-3^ | average | Glorot uniform | True | 0.873953 |
| U-Net++  (2,8,2) | (3, 3, 3) | 8 | True | swish | 0.2 | 8 | Adam | 10^-3^ | average | Glorot normal | True | 0.774147 |
| U-Net++  (2,8,2) | (3, 3, 3) | 32 | True | GELU | 0 | 8 | Adam | 10^-3^ | max | Glorot normal | True | 0.92649 |
| **U-Net++**  **(2,8,2)** | **(3,3,3)** | **16** | **True** | **ELU** | **0** | **8** | **Adam** | **10^-2^** | **max** | **Glorot normal** | **True** | **0.923** |
| U-Net++  (2,8,2) | (3, 3, 3) | 16 | True | ELU | 0.4 | 32 | Adam | 10^-3^ | average | random normal | True | 0.873313 |
| U-Net  (3,4,2) | (2, 2, 2) | 32 | True | GELU | 0.2 | 32 | Adam | 10^-3^ | average | Glorot uniform | True | 0.55943 |
| U-Net  (3,4,2) | (2, 2, 2) | 16 | True | GELU | 0.4 | 32 | Adam | 10^-2^ | average | Glorot uniform | True | 0.14882 |
| U-Net  (3,4,2) | (3, 3, 3) | 16 | True | SELU | 0.2 | 32 | Adam | 10^-2^ | max | Glorot uniform | True | 0.68446 |
| U-Net  (3,4,2) | (5, 5, 5) | 32 | True | GELU | 0.4 | 16 | Adam | 10^-2^ | average | Glorot uniform | True | 0.63995 |
| **U-Net**  **(3,4,2)** | **(3,3,3)** | **16** | **True** | **ELU** | **0** | **8** | **Adam** | **10^-2^** | **max** | **Glorot uniform** | **True** | **0.94318** |
| U-Net  (3,4,2) | (3, 3, 3) | 16 | True | ELU | 0 | 8 | Adam | 10^-2^ | max | Glorot normal | True | 0.9269 |
| U-Net  (3,4,2) | (5, 5, 5) | 8 | True | GELU | 0.2 | 8 | Adam | 10^-2^ | max | random normal | True | 0.91256 |
| V-Net  (3,2,3) | (3, 3, 3) | 8 | True | GELU | 0.2 | 8 | Adam | 10^-2^ | average | random normal | True | 0.937 |
| V-Net  (3,2,3) | (3, 3, 3) | 16 | True | swish | 0 | 32 | Adam | 10^-3^ | average | Glorot normal | True | 0.9373 |
| V-Net  (3,2,3) | (5, 5, 5) | 8 | True | GELU | 0.4 | 16 | Adam | 10^-3^ | max | Glorot normal | True | 0.89056 |
| V-Net  (3,2,3) | (3, 3, 3) | 16 | True | GELU | 0.2 | 32 | Adam | 10^-3^ | average | Glorot uniform | True | 0.88285 |
| **V-Net**  **(3,2,3)** | **(3,3,3)** | **16** | **True** | **ELU** | **0** | **8** | **Adam** | **10^-2^** | **max** | **Glorot uniform** | **True** | **0.945** |
| V-Net  (3,2,3) | (3, 3, 3) | 32 | True | swish | 0 | 8 | Adam | 10^-2^ | max | Glorot uniform | True | 0.943 |

*(continuation)*

| **architecture**  **(blocks, layers, dimensions)** | **kernel size** | **number of filters** | **renormalisation** | **activation function** | **dropout** | **batch size** | **optimiser** | **learnrate** | **pooling** | **kernel initialiser** | **deconvolution** | **DSC** |
| --- | --- | --- | --- | --- | --- | --- | --- | --- | --- | --- | --- | --- |
| Attention U-Net  (3,2,3) | (3, 3, 3) | 16 | True | GELU | 0.2 | 16 | Adam | 10^-2^ | max | Glorot uniform | False | 0.92586 |
| Attention U-Net  (3,2,3) | (3, 3, 3) | 32 | True | SELU | 0.4 | 32 | Adam | 10^-2^ | max | Glorot normal | False | 0.59272 |
| Attention U-Net  (3,2,3) | (3, 3, 3) | 8 | True | GELU | 0.4 | 16 | Adam | 10^-3^ | average | random normal | True | 0.37835 |
| **Attention U-Net**  **(3,2,3)** | **(5, 5, 5)** | **16** | **True** | **swish** | **0.2** | **8** | **Adam** | **10^-2^** | **average** | **random normal** | **True** | **0.936** |
| Attention U-Net  (3,2,3) | (5, 5, 5) | 32 | True | ELU | 0.2 | 16 | Adam | 10^-3^ | average | Glorot uniform | False | 0.85055 |

**^a^** The model with the highest DSC in each of the architectures tried is highlighted in bold

**Attention U-Net**

The top skip connection in the Attention U-Net both for 1MSA and 2MSA attention coefficients are showed in **Figure 6**. For both approaches, a trained Attention U-Net is capable of placing more emphasis on the ROI than the milieu comprised of surrounding features, thereby reducing the influence of artefacts and background effects. Nonetheless, it was noticed that they followed different focussing patterns. 1MSA learned to put emphasis on the whole myocardial region both for systole and diastole phases. In contrast, 2MSA only followed this pattern for the diastole phase and for the systole phase only attention coefficients with high values are found in the myocardium contour.


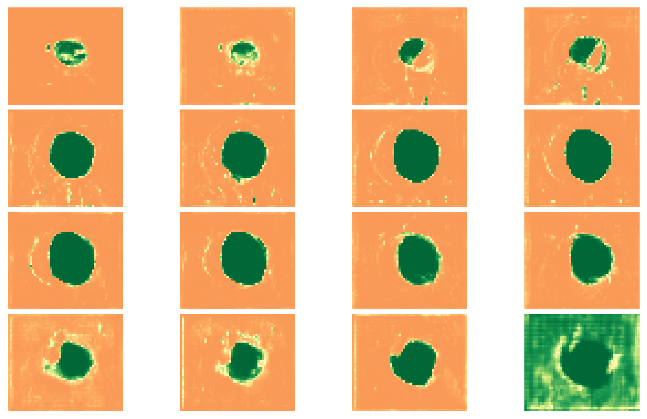

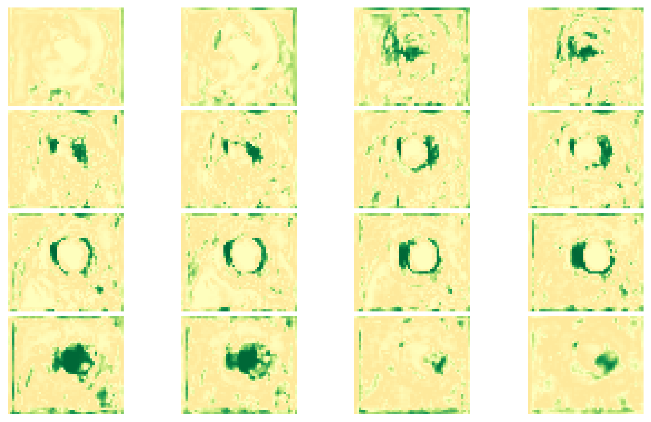


**C**

**B**

**A**

**D**


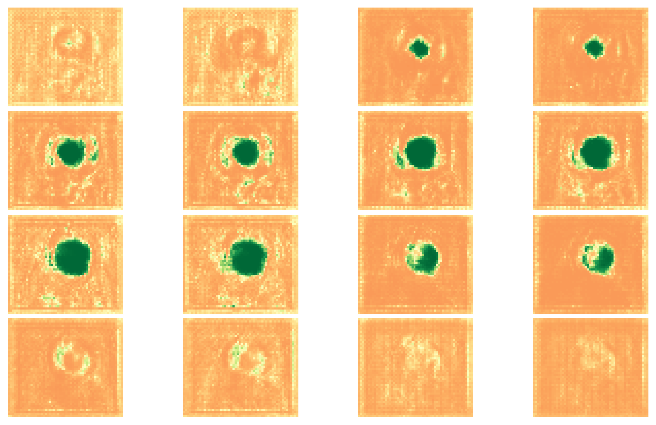

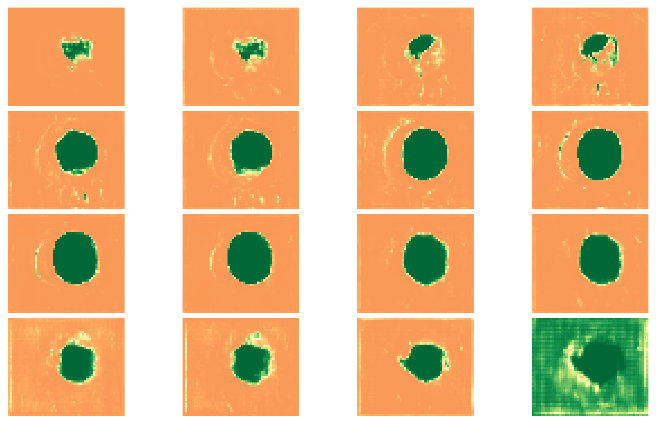


**Figure S6**. Attention coefficient distribution for example images from Study 5.

**A**: 2MSA diastole phase. **B**: 2MSA systole phase. **C**: 1MSA diastole phase. **D**: 1MSA systole phase.

1. **Interoperator Volume Agreement**

A close inspection of **Figure S7**, reveals a strong correlation between the analysed operators. However, it can be easily noticed that Operator 2 tends to generate segmentations which lead to volume underestimations if compared to Operator 1. Indeed, this is in line with previous observations made by closely examining the LOESS fit from **Figure S1**. Interestingly, the diastolic segmentations seem to have a higher degree of agreement than the systolic segmentations.


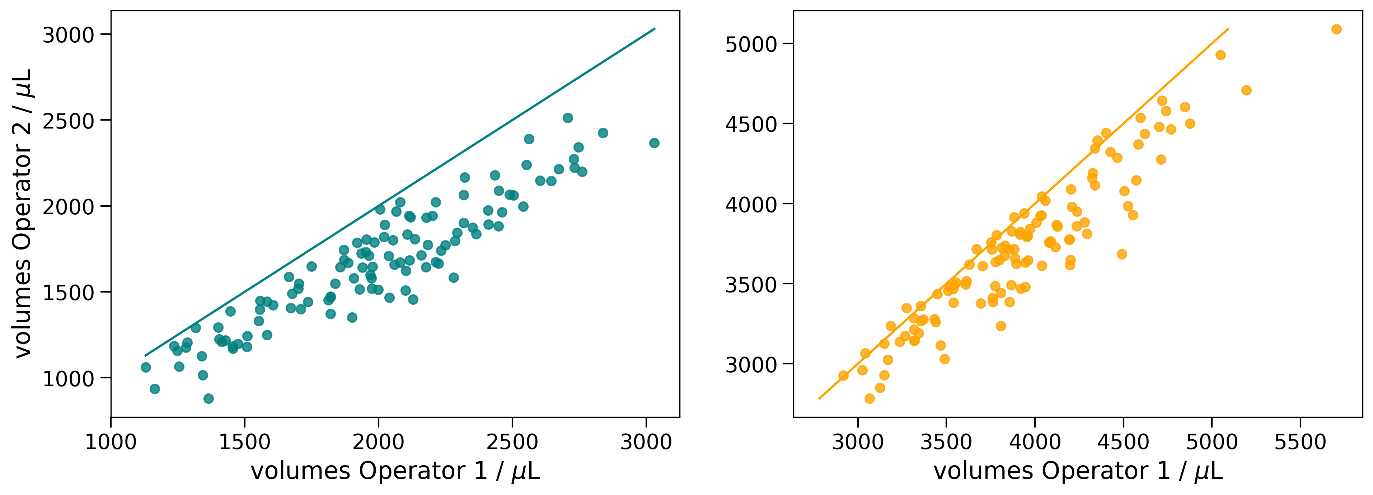


**B**

**A**

**Figure S7.** Correlation plot between operators. A solid line indicates a perfect correlation.

**A**: Systole volume estimation (ρ = 0.93)

**B**: Diastole volume estimation (ρ = 0.93)

1. **Bland-Altman Plots for Automation Feasibility Studies**


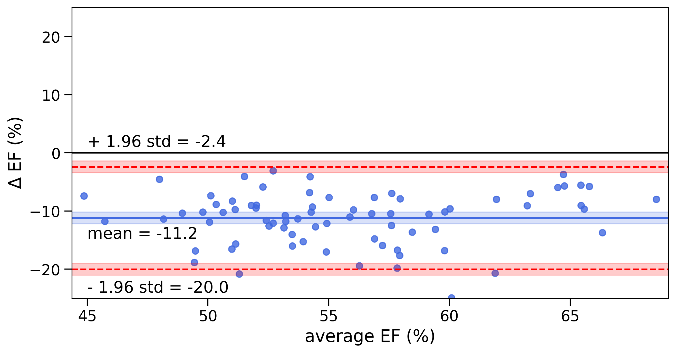

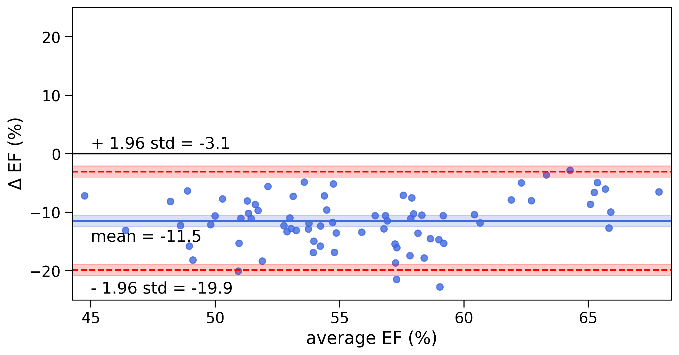


**A**

**B**


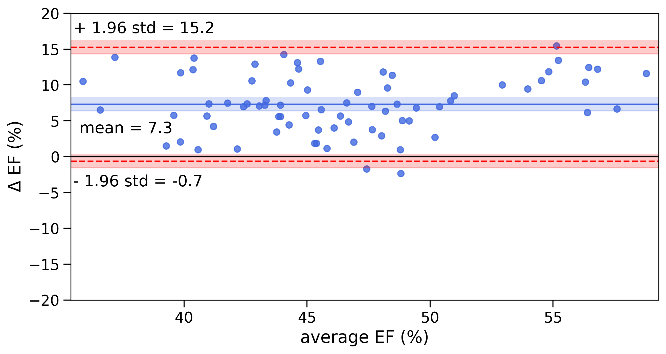

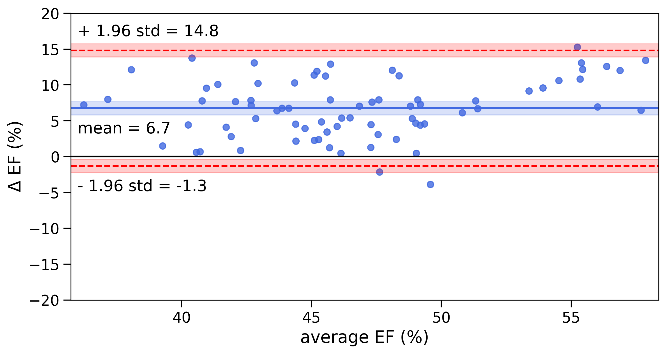


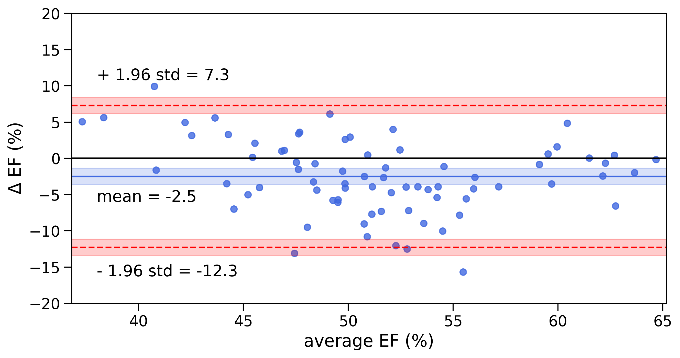

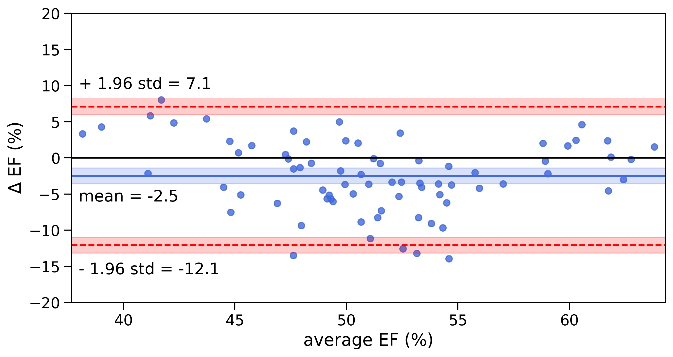


**C**

**Figure S8.** Bland-Altman plots for agreement analysis by selection parameter. The bias is marked with a continuous blue line (**─**) and the equality line is marked with a continuous black line (**─**). The upper 95% confidence interval bound is marked with a discontinuous green line (--) and the lower one with a discontinuous red line (--). Both are shaded with their respective 95% confidence interval bounds.

**A**: Heart volume as selection parameter. Agreement for polynomial fit displays a bias of -11.5 ± 4.3 % EF (left) and agreement for GP fit displays a bias of -11.2 ± 4.3 % EF (right).

**B**: Surface area as selection parameter. Agreement for polynomial fit displays a bias of 6.7 ± 4.1 % EF (left) and agreement for GP fit displays a bias of 7.4 ± 4.3 % EF (right).

**C**: Midslice area as selection parameter. Agreement for polynomial fit displays a bias of -2.5 ± 4.9 % EF (left) and agreement for GP fit displays a bias of -2.5 ± 5.2 % EF (right).

1. **Implementation Details**

Herein we show a schematic representation of the used architectures taken from their original publications.^1-4^ We refer the interested readers to the original manuscripts for more details.

- 1. **U-Net^1^**

**Figure S7** displays a schematic representation of the popular U-Net architecture. Blue boxes represent multi-channel feature maps with the number of channels denoted above the box and x and y dimensions at the lower left edge. White boxes denote the copied feature maps. Note that in our project we used a 3D extension of the original U-Net by using 3D-variants of convolutional, downsampling, padding, and up-convolutional layers.


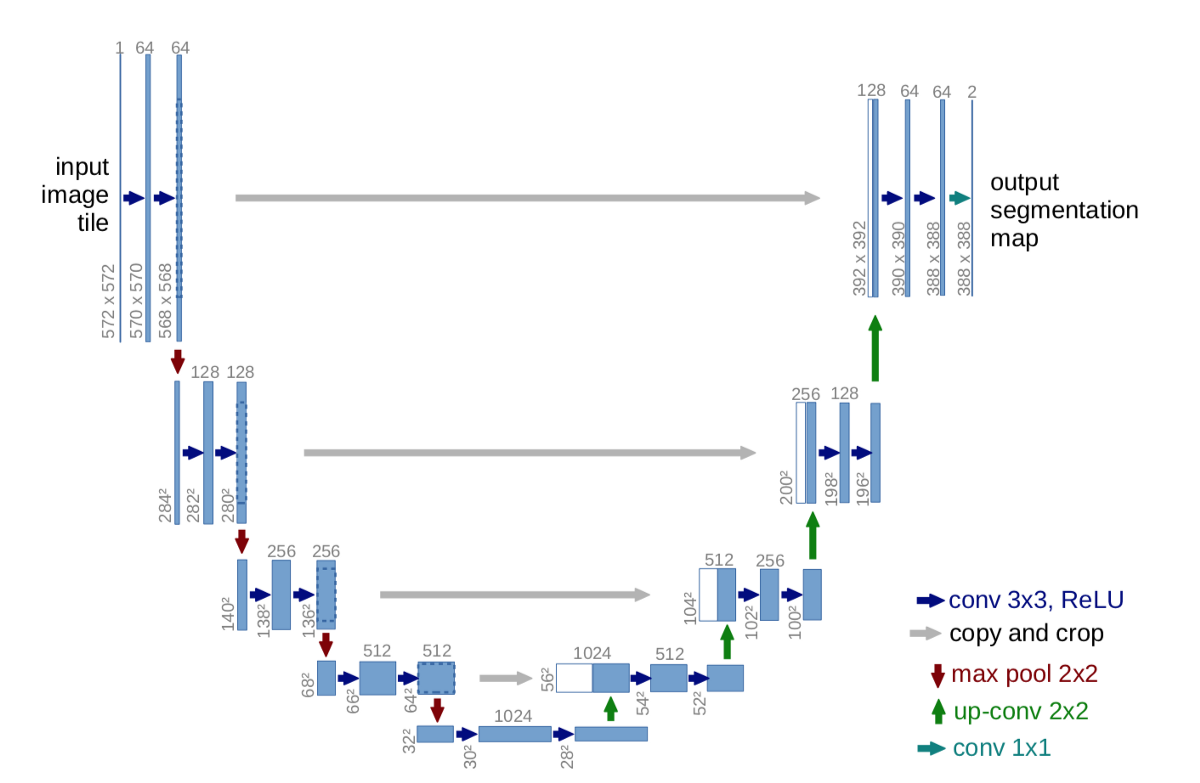


**Figure S7.** U-Net architecture diagram from Ronneberger et al.^1^

- 1. **Attention U-Net^2^**

In **Figure S8** a schematic representation of the Attention U-Net is displayed. Similarly to the U-Net, the architecture contains the usual downsampling and upsampling blocks Additionally, attention gates are used after the skip connections to propagate the features. Such gating of the contextual information extracted in coarser scales aims to achieve improved features selectivity, i.e. spatial region selection, and by extension the segmentation results.


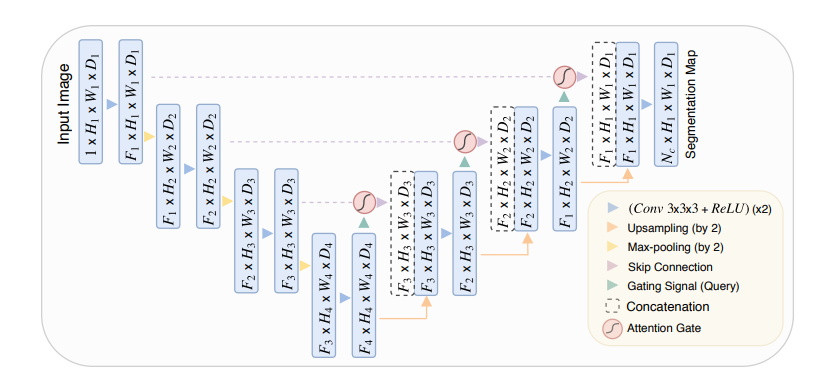


***a***


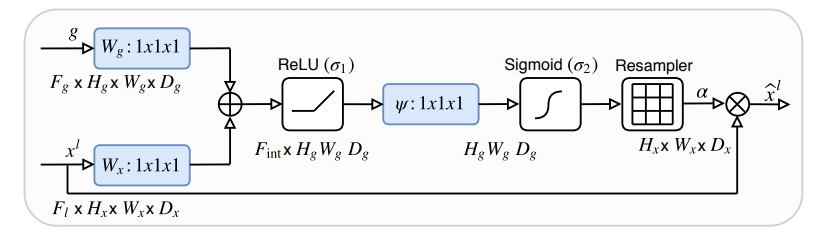


***b***

**Figure S8.** Attention U-Net architecture diagram (a) and detail of attention gates (b) from *Oktay et al.*^2^

- 1. **U-Net++^3^**

**Figure S9** shows a simplified diagram of the U-Net++. Each node represents a convolutional block. The architecture again containing the traditional downsampling and upsampling steps (downward and upward arrows) and skip connections (dot arrows). Additionally, dense skip connections are added at the level of skip connections to enable dense feature propagation and therefore a more flexible feature fusion at each node.


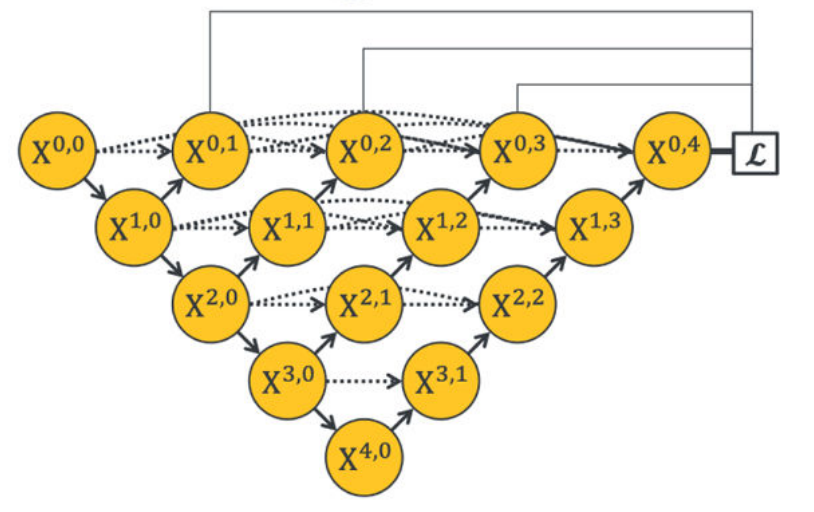


**Figure S9.** U-Net++ architecture diagram from *Zhou et al.*^3^

- 1. **V-Net^4^**

**Figure S10** is a schematic representation of V-Net. As an improvement to the original U-Net, V-Net was specifically designed to process 3D volumes in medical contexts.


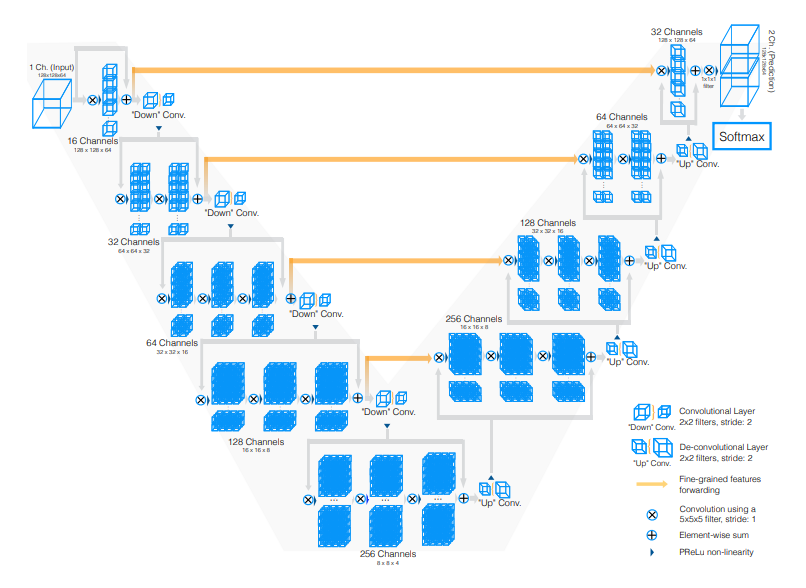


**Figure S11.** V-Net architecture diagram from *Milletari et al*.^4^

**References**

1. Ronneberger, O., Fischer, P. & Brox, T. U-Net: Convolutional Networks for Biomedical Image Segmentation. Lect. Notes Comput. Sci. (including Subser. Lect. Notes Artif. Intell. Lect. Notes Bioinformatics) 9351, 234–241 (2015).
2. Oktay, O. et al. Attention U-Net: Learning Where to Look for the Pancreas. (2018).
3. Zhou, Z., Siddiquee, M. M. R., Tajbakhsh, N. & Liang, J. UNet++: Redesigning Skip Connections to Exploit Multiscale Features in Image Segmentation. IEEE Trans. Med. Imaging 39, 1856–1867 (2020).
4. Milletari, F., Navab, N. & Ahmadi, S. A. V-Net: Fully convolutional neural networks for volumetric medical image segmentation. Proc. - 2016 4th Int. Conf. 3D Vision, 3DV 2016 565–571 (2016) doi:10.1109/3DV.2016.79.
